# Supplementary material for: Ubiquitous short-range order in multi-principal element alloys
Source: Nat Commun. 2024 Aug 1;15:6486. doi: 10.1038/s41467-024-49606-1 (PMC11294451; doi:10.1038/s41467-024-49606-1)
Supplement: Supplementary file 1 — Supplementary Information [file 41467_2024_49606_MOESM1_ESM.pdf]

Supplementary Materials

Ubiquitous short-range order in multi-principal element alloys

Ying Han[1,†], Hangman Chen[2,†], Yongwen Sun[1], Jian Liu[3], Shaolou Wei[4,#], Bijun Xie[2], Zhiyu Zhang[1], Yingxin Zhu[1], Meng Li[5], Judith Yang[5,6], Wen Chen[3], Penghui Cao[2,*], Yang Yang[1,*]

[1] Department of Engineering Science and Mechanics and Materials Research Institute, The Pennsylvania State University, University Park, PA, 16802, USA.
[2] Department of Mechanical and Aerospace Engineering, University of California, Irvine, CA 92697, USA.
[3] Department of Mechanical and Industrial Engineering, University of Massachusetts, Amherst, MA 01003, USA.
[4] Department of Materials Science and Engineering, Massachusetts Institute of Technology, Cambridge, MA 02139, USA.
[5] Department of Petroleum and Chemical Engineering, University of Pittsburgh, Pittsburgh, PA 15261, USA.
[6] Center for Functional Nanomaterials, Brookhaven National Laboratory, Upton, NY 11973, USA.

[†] These authors contributed equally to this work.
[#] Currently at: Max-Planck-Institut für Eisenforschung, 40237, Düsseldorf, Germany.
[*] Email of corresponding authors: caoph@uci.edu; yangyang@alum.mit.edu

Table of Contents

SUPPLEMENTARY TABLES 3

SUPPLEMENTARY NOTE 1 12

SUPPLEMENTARY NOTE 2 13

SUPPLEMENTARY NOTE 3 14

SUPPLEMENTARY NOTE 4 15

SUPPLEMENTARY NOTE 5 16

SUPPLEMENTARY NOTE 6 17

SUPPLEMENTARY NOTE 7 18

SUPPLEMENTARY NOTE 8 19

SUPPLEMENTARY FIGURE 20

SUPPLEMENTARY REFERENCES 52

Supplementary Tables

Supplementary Table 1. Overview of literature regarding the adjustment of SRO via heat treatment and change of cooling rate.

Year	Materials	Publication title	Thermal treatments
2017	CoCrNi	Local structure and short-range order in a NiCoCr solid solution alloy	Homogenized at 1,200 °C for 24 h in vacuum and then annealed at 800 °C for 1 h in air.
2017	CoCrNi	Friction stress and Hall-Petch relationship in CoCrNi equiatomic medium entropy alloy processed by severe plastic deformation and subsequent annealing	Homogenized at 1,200 °C for 12 h in vacuum, went through high-pressure torsion and annealed at various temperatures ranging from 600 °C to 1000 °C for 30 s to 1800 s.
2017	CoCrNi based MEA	Heterogeneous precipitation behavior and stacking-fault-mediated deformation in a CoCrNi-based medium-entropy alloy	Solution-treated at 1200 °C in air for 2 h and water quenched followed by cold rolling for a ~66% thickness reduction. Then annealed at 1160 °C in air for 3 min then water quenched. Recrystallized samples were isothermally aged at 800 °C for 2 h and water quenched.
2017	CoCrNi	The evolution of the deformation substructure in a Ni-Co-Cr equiatomic solid solution alloy	Homogenized at 1,473 K for 24 h and water quenched. Then cold rolled and recrystallized at 1,198 K for 1 h.
2017	CoCrFeMnNi	Thermally activated deformation and the rate controlling mechanism in CoCrFeMnNi high entropy alloy	Homogenized at 1,100°C for 6h in Ar followed by cold rolling to achieve a 50% thickness reduction. Then annealed at 800°C for 1 h in Ar.
2018	CoCrNi, CoCrFeNi and CoCrFeMnNi	Local lattice distortion in NiCoCr, FeCoNiCr and FeCoNiCrMn concentrated alloys investigated by synchrotron X-ray diffraction	Homogenized at 1,473 K for 24 h, and cold-rolled to obtain a ~86% thickness reduction. Then annealed at 1,073 K for 1 h.
2018	CoCrNi	Influence of deformation induced nanoscale twinning and FCC-HCP transformation on hardening and texture development in medium entropy CrCoNi alloy	Homogenized for 24 h at 1,200 °C and cold-rolled to get a 70% thickness reduction. Then annealed in air at 900 °C for 1 h.
2018	CoNiV	Ultrastrong medium-entropy single-phase alloys designed via severe lattice distortion	Homogenized at 1,200 °C for 24 h and cold-rolled to achieve a 75% thickness reduction. Then recrystallized at 900 °C for 60 min followed by water quenching.
2018	CoCrNi	On the mechanical response and microstructure evolution of NiCoCr single crystalline medium entropy alloys	Homogenized at 1,473 K for 24 h and then water quenched.
2019	Ti_6Al	Direct imaging of short-range order and its impact on deformation in Ti-6Al	Heat-treated at 965 °C for 2 h and some samples were aged at 420 °C for 1 week to promote the SRO.
2019	CoCrFeMnNi and CoCrFeNiPd	Tuning element distribution, structure and properties by composition in high-entropy alloys	Homogenized in vacuum at 1,200 °C for 24 h then rolled into a 1.8 mm plates from 12.7 mm at room temperature. Recrystallized at 1,150 °C for 1 h or 20 min in vacuum to get different samples.
2019	CoCrFeMnNi	Strain-rate sensitivity of high-entropy alloys and its significance in deformation	Homogenized at 1,100 °C for 6h in Ar followed by cold rolling to achieve a 50% thickness reduction. Then annealed at 800 °C for 1 h in Ar.
2019	$Ni_{20}Cr_{3.2}Al_{2.5}Fe$	Short-range ordering in a commercial Ni-Cr-Al-Fe precision resistance alloy	Homogenized at 1,323 K for 2 h and then water quenched. Some samples were aged at 748 K for 50 h.
2020	CoCrNi	Short-range order and its impact on the CrCoNi medium-entropy alloy	Homogenized at 1,200 °C for 48 h then water quenched to room temperature or homogenized at 1,200 °C for 48 h then aged at 1,000 °C for 120 h followed by furnace cooling.
2020	CoCrFeNi	Simultaneous enhancement of strength and ductility in a NiCoCrFe high-entropy alloy upon dynamic tension: Micromechanism and constitutive modeling	Annealed at 1,173 K for 1 h and water quenched. (No intermediate annealing between cold-forging and cold-rolling treatments was conducted.)

2020	$Al_{8}Co_{17}Cr_{17}Cu_{8}Fe_{17}Ni_{33}$	Short-range chemical order and local lattice distortion in a compositionally complex alloy	Homogenized at 1,250 °C for 1 h under Ar and then recrystallized at temperatures of 700, 800, 900, 1,000, 1,100 and 1,200 °C for 1 h.
2021	CoNiV	Direct observation of chemical short-range order in a medium-entropy alloy	Homogenized in vacuum at 1,373 K for 2 h followed by water quenching. Then cold rolled to achieve a 90% thickness reduction and annealed at 1,173 K for 150 s.
2021	CoCrFeNi	Understanding chemical short-range ordering/demixing coupled with lattice distortion in solid solution high entropy alloys	Selective laser melting then annealed at 800 °C for 168 h.
2021	CoCrFeMn	Chemical short-range order in Fe50Mn30Co10Cr10 high-entropy alloy	Homogenized at 1,150 °C for 24 h and water quenched. Then hot rolled for 85% thickness reduction and cold rolled for 30 % thickness reduction. Recrystallized at 760 °C for 10 min or 600 °C for 1 h.
2021	CoCrFeMnNi	Effects of annealing on hardness, yield strength and dislocation structure in single crystals of the equiatomic Cr-Mn-Fe-Co-Ni high entropy alloy	HEA grown in furnace, annealed at 1,200 °C for one week and water quenched. Re-annealed at 900, 1,000 and 1,100 °C for one week and water quenched.
2021	CoCrFeNi and CoCrFeMnNi	Bimodality of incipient plastic strength in face-centered cubic high-entropy alloys	Homogenized at 1,000 and 1,100 °C for 1 h respectively for different components followed by water quenching, and aged at 900 °C for 24 h followed by slow furnace cooling.
2021	HfNbTiZr	Local chemical fluctuation mediated ductility in body-centered-cubic high-entropy alloys	As cast alloys
2021	CoCrNi	Direct observation of local chemical ordering in a few nanometers range in CoCrNi medium-entropy alloy by atom probe tomography and its impact on mechanical properties	Cold rolled to achieve a 30% thickness reduction and homogenized at 1,100 °C for 24 h. Annealed one is at 700 °C for 384 h followed by water quenching.
2021	CoCrNi	Element dependence of local disorder in medium-entropy alloy CrCoNi	Homogenized at 1,473 K for 24 h. Different heat treatments were conducted, including 1,473 K for 5 min, 1,123 K for 2 h and 773 K for 2 h.
2021	$(CoCrFeNi)_{94}Ti_{2}A_{l4}$	Microstructure and nanomechanical behavior of an additively manufactured $(CrCoNiFe)_{94}Ti_{2}Al_{4}$ high-entropy alloy	LENS printed and some samples underwent heat treatment at 650 °C for 4 h.
2022	CoCrNi	Atomic-scale evidence of chemical short-range order in CrCoNi medium-entropy alloy	Homogenized in vacuum at 1,100 °C for 12 h, rapid water quenched, cold-rolled and annealed at 600 °C or 1,000 °C for 1 h.
2022	CoCrNi	Determination of peak ordering in the CrCoNi medium-entropy alloy via nanoindentation	Homogenized in vacuum at 1,200 °C for 24 h and cold rolled to achieve a ~76% thickness reduction. Recrystallized at 1,000 °C for 0.5 h followed by ice water quenching. Then aged at 600 °C, 700 °C, 800 °C, 900 °C, and 1,000 °C for 240 h and splat quenched.
2022	CoCrFeMnNi	Electrical resistivity and short-range order in rapid-quenched CrMnFeCoNi high-entropy alloy	Rapidly quenched (RQ-) CoCrFeMnNi alloy prepared by the melt spinning technique using a rotating single copper wheel in Ar and then annealed at 1023 K for 1 h.
2022	CrFeNi	In situ neutron diffraction unravels deformation mechanisms of a strong and ductile FeCrNi medium entropy alloy	Homogenized at 1,200 °C for 1 h, then performed hot extrusion immediately (7:1) and cooled down in the air.
2022	CoCrNi	Atomic-scale evidence of chemical short-range order in CrCoNi medium-entropy alloy	Homogenized at 1,100 °C for 12 h and then water quenched. A 90% thickness reduction was achieved by cold rolling, followed by annealing at 600 °C and 1,000 °C for 1 h.
2022	CoNiV	Structure motif of chemical short-range order in a medium-entropy alloy	Homogenized at 1,100 °C for 2 h and then water quenched. Cold rolled to achieve a 90% thickness reduction (1 mm thickness) followed by annealing at 880 °C for 3 min.
2022	CoCrNi	Data-driven electron-diffraction approach reveals local short-range ordering in CrCoNi with ordering effects	Hot forged at 1,200 °C and hot rolled at 105 °C for a 50 % thickness reduction. Then homogenized at 1,200 °C for 2 h in Ar. Some samples multipassed cold rolling and annealing (750 °C and 900 °C for 3 min) and water quenched. Some samples were annealed at 900 °C for 3 min.
2022	$Al_{0.3}CoCrFeNi$	Thermal physical properties of high entropy alloy $Al_{0.3}CoCrFeNi$ at elevated temperatures	Melt powders at above 1,500 °C followed by water quenching.
2022	CoCrNi	Effects of cold-rolling and subsequent annealing on the nano-mechanical and creep behaviors of CrCoNi medium-entropy alloy	Homogenized at 1,000 °C for 6 h in Ar and then cold rolled to achieve a 70% thickness reduction. Different annealings were conducted at 600 °C, 700 °C, and 1100 °C for 1 h.

2023	CoCrNi	Evolution of short-range order and its effects on the plastic deformation behavior of single crystals of the equiatomic Cr-Co-Ni medium-entropy alloy	Homogenized at 1,473 K for 168 h followed by cold rolling for a 50% thickness reduction. Then recrystallized at 1,273 K for 2 h followed by water quenching. Isothermally annealed for various times at temperatures ranging from 573 to 973 K. Single crystals were grown with an optical floating-zone furnace in Ar with a 10 mm/h growth rate. This sample was homogenized at 1,472 K for 168 h and water quenched. Then, they underwent annealing at 573 to 873 K for 168 to 504 h and water quenching.
2023	CoFeNiV	Formation of chemical short-range orders of two kinds and the co-existence with medium-range orders in an equiatomic VFeCoNi alloy	Homogenized at 1,100 °C for 2 h and cold rolled from 12 mm to 1 mm thickness. Recrystallized at 800 °C for 15 min followed by water quenching.
2023	CoMnNi and CoCrNi	Time-temperature dependent short- and long-range structural transformation in medium-entropy alloys	Annealed at 1,273 K to 1,373 K for 2 h and quenched to ambient temperature. Further annealing was performed at 473, 673, 873 and 1073 K under Ar for different time periods.
2023	CoNiV	Characterization of chemical short-range order in VCoNi medium-entropy alloy processed by spark plasma sintering	Ball milled for 8 h of CoNiV Powders and mechanically alloyed powders were compacted and consolidated via spark plasma sintering at 1,000 °C for 10 min with a uniaxial pressure of 40 MPa. Then hot rolled at 1,000 °C to obtain a 50% thickness reduction.
2023	CoCrNi	Formation condition and effect on the early stages of plastic deformation of chemical short-range order in Cr-Co-Ni medium-entropy alloy	Homogenized at 1473 K and hot rolled to reduce 90% thickness. Then, heat treated at 673 K for 2 or 10 days. (Heated with constant rate of 1 K min^{-1} and cooled with constant rates from 0.1 to 30 K min^{-1})
2023	$Fe_{46.8}Mn_{30}Co_{10}Cr_{10}N_{3.2}$ and $Fe_{61.4}Mn_{16.5}Cr_{17.9}Mo_{1.3}N_{2.9}$	Interstitial-driven local chemical order enables ultrastrong face-centered cubic multicomponent alloys	Hot forged at 1,200 °C and hot rolled at 105 °C for a 50% thickness reduction. Then homogenized at 1,200 °C for 2 h in Ar. Some samples multipassed cold rolling and annealing (750 °C and 900 °C for 3 min) and water quenched. Some samples were annealed at 900 °C for 3 min.

Supplementary Table 2. Comparison of different SRO characterization methods.

Methods	HR-STEM	Resistometry	EF-SAED	Atomic EDS	X-ray / neutron scattering	APT	AET	ESQ-SAED (This work)
Larger area characterization	○	●	●	○	●	○	○	●
Time-efficient	○	●	●	○	○	○	○	●
Insensitive to impurity	●	○	●	○	●	○	○	●
Quantitative	◑	◑	○	◑	○	◑	●	◑
Insensitive to focus and sample thickness	○	●	●	○	●	N/A	○	●
Experimental convenience	○	●	●	○	○	○	○	●

Supplementary Table 3. The effect of annealing temperature on the formation of CSRO.

Temperature	Type of paring	$(\overline{\Delta\alpha})_{\text{Solidified}}$	$(\overline{\Delta\alpha})_{\text{Annealed}}$	$(\overline{\Delta\alpha})_{\text{Annealed}} - (\overline{\Delta\alpha})_{\text{Solidified}}$	$((\overline{\Delta\alpha})_{\text{Annealed}} - (\overline{\Delta\alpha})_{\text{Solidified}})/(\overline{\Delta\alpha})_{\text{Solidified}} \times 100$ %
**1,315 K**	Ni-Ni	0.71	0.78	0.07	9.90%
	Co-Cr	0.95	1.04	0.09	9.50%
	Ni-Co	-0.4	-0.5	-0.1	-25.00%
	Cr-Cr	-0.63	-0.8	-0.17	-27.00%
	Ni-Cr	-0.31	-0.25	0.06	19.40%
	Co-Co	-0.56	-0.55	0.01	1.80%
**1,015 K**	Ni-Ni	0.67	1.14	0.47	70.10%
	Co-Cr	0.86	1.28	0.42	48.80%
	Ni-Co	-0.43	-0.77	-0.34	-79.10%
	Cr-Cr	-0.61	-0.92	-0.31	-50.80%
	Ni-Cr	-0.39	-0.37	0.02	5.10%
	Co-Co	-0.6	-0.52	0.08	13.30%

Supplementary Table 4. Pair cohesive energy and pairwise order parameters.

**Pair**	**Cohesive energy (eV/atom)**	**Pairwise order parameter**
Ni-Ni	-4.45 (lowest)	**0.78 (second highest)**
Co-Co	-4.41	-0.55
Cr-Cr	-3.89	-0.63
Ni-Co	-4.40	-0.50
Ni-Cr	-4.25	-0.25
Co-Cr	-4.24	**1.04 (highest)**

Supplementary Table 5. The heat of mixing of binary random alloy.

Binary random alloy	Heat of mixing (eV)
Ni-Co	0.0256
Ni-Cr	-0.0752
Co-Cr	-0.0952

Supplementary Table 6. The heat of mixing of binary random alloy using Choi EAM potential.

**Binary random alloy**	**Heat of mixing (eV)**
Ni-Co	-0.0176
Ni-Cr	0.0009
Co-Cr	0.0427

Supplementary Table 7. Summary of the parameters used for the sample fabrication by LPBF and LDED.

Alloys	CoCrNi		CoCrFeNi		CoCrFeMnNi	
Parameters	LPBF	LDED	LPBF	LDED	LPBF	LDED
laser power P (W)	370	200	370	220	300	250
Scanning speed v (mm s^{-1})	800	12.5	800	12.5	1000	12.5
Hatch spacing s (mm)	0.08	0.4	0.08	0.4	0.08	0.4
Layer thickness h (mm)	0.04	0.25	0.04	0.25	0.04	0.35
Scan rotation (◦)	90	90	90	90	90	90

Supplementary Note 1

The modified order parameter used in this manuscript is $\Delta\alpha_{i\text{-}j} = N_{i\text{-}j} - N_{i\text{-}j,0}$, where $N_{i\text{-}j}$ is the actual number of i-j pairs in the first nearest neighbour and $N_{i\text{-}j,0}$ is the number of i-j pairs for the random solid solution. A positive $\Delta\alpha_{i\text{-}j}$ indicates a favoured and increased number of pairs, meaning that element i tends to bond with element j, while a negative value represents an unfavoured pairing. Therefore, the value of $\Delta\alpha_{i\text{-}j}$ directly quantifies the number of i-j pairs changed from random mixing. For example, $\Delta\alpha_{Ni-Ni} = 2$ indicates there are two extra Ni-Ni pairs when comparing with random solid solution.

We have also computed the more well-known Warren-Cowley parameter for comparison. The Warren-Cowley parameter for multicomponent systems in the following formula[1-3]:

$$\alpha_m^{ij} = \frac{P_m^{ij} - c_j}{\delta^{ij} - c_j} \tag{1}$$

where m means the mth nearest neighbouring shell, P_m^{ij} is the probability of finding a j-type atom around the i-type atom in the mth shell, δ^{ij} is the Kronecker delta function, and c_j is the average concentration of j-type atom in the system. It is noted that for the same type, $i=j$ (i.e., $\delta^{ij}=1$), a positive α_m^{ij} suggests the tendency of segregation. When $i \neq j$ ($\delta^{ij}=0$), a negative α_m^{ij} suggests the tendency of segregation.

In **Supplementary Fig. 15**, we show the comparison of these two kinds of order parameters, namely modified parameter $\Delta\alpha_{i\text{-}j}$ and Warren-Cowley order parameter $\alpha_{m=1}^{ij}$. As can be seen, the spatial distributions of order parameters from the two calculations exhibit essentially the same characteristics. It is worth noting that the Warren-Cowley order parameters, ranging from -2 to 1, can be positive (Ni-Ni) or negative (Co-Cr) even for the same ordering (segregation). The results indicate that the parameter $\Delta\alpha_{i\text{-}j}$, carrying the essential feature of the Warren-Cowley parameter, more straightforwardly reflects the degree of local chemical ordering.

Supplementary Note 2

To investigate the influence of the cooling rate on the CSRO formation, we further simulate the solidification at 1,015 K with a cooling rate of 10^{11} K s^{-1}. The distributions of atoms and local pairwise order parameters (**Supplementary Fig. 18a**) suggest that even with a cooling rate of 10^{11} K s^{-1}, CSRO can still form. The average $\Delta\alpha_{Ni\text{-}Ni}$ and $\Delta\alpha_{Co\text{-}Cr}$ are 0.67 and 0.86, respectively (**Supplementary Fig.18e**), which are smaller compared to those obtained using a cooling rate of 10^{10} K s^{-1} (**Fig. 3**). This suggests that the degree of CSRO decreases with a rising cooling rate. On the other hand, long-time annealing at 1,015 K significantly enhances the CSRO, as revealed by the distribution of local pairwise order parameters (**Supplementary Fig. 18e**). To scrutinize the effect of annealing temperature on the formation of CSRO, we have computed the disparities of local pairwise order parameters between the as-solidified and as-annealed states at 1,015 K and 1,315 K, as shown in **Supplementary Table 3**. Taking the local Ni-Ni paring in the first nearest neighbor shell as an example, the average value of $\Delta\alpha_{Ni\text{-}Ni}$ receives a 70.1 % increase through annealing at 1,015 K, while this trend is imperceptible (only ~ 9.9 %) at an annealing temperature of 1,315 K. In addition, the average values of $\Delta\alpha_{Ni\text{-}Ni}$ and $\Delta\alpha_{Co\text{-}Cr}$ in the as-annealed system at 1,015 K are 1.14 and 1.28, respectively (**Supplementary Fig. 18e** and **Supplementary Table 3**), which are even higher than that at 1,315 K (**Fig. 3g** and **Supplementary Table 3**). The more pronounced CSRO at a lower annealing temperature can be attributed to the increasing role of enthalpic contribution to the total free energy at a lower annealing temperature[1]. In addition, we observed that the transition zone from the disordered substrate to the ordered solidified zone at 1,015 K is also narrow (**Supplementary Fig. 18f**). Despite the varying degrees of CSRO in the solidified regions under different cooling rates, the presence of similar narrow transition zones suggests that the formation of CSRO is inevitable during the solidification of CoCrNi MEA.

Furthermore, we compared the crystal growth velocity and diffusivities of atoms in liquid at 1,015 K to understand how the cooling rate alters the degree of CSRO. The average growth velocity is 27.93 nm ns^{-1} (**Supplementary Fig. 19a**), showing an increase of 66.17% compared to that at 1,315 K. The diffusivities of Ni, Co, and Cr are estimated at 0.61, 0.51, and 0.44 nm^2 ns^{-1}, respectively (**Supplementary Fig. 19b**), corresponding to a decrease of 70.39%, 72.58%, and 73.33%, respectively compared to 1,315 K. The diffusion distances of Ni, Co, and Cr, therefore, decrease by 67.86%, 69.23%, and 72% to 0.09, 0.08, and 0.07 nm, respectively (**Supplementary Fig. 19c**) when the solidification front advances 0.35 nm. The observation that the diffusion distances are notably less than 0.35 nm indicates that the supercooled liquid's inadequate diffusion speed, caused by a high cooling rate, limits the local atomic reconfiguration necessary for forming preferred or un-preferred atomic pairs.

Supplementary Note 3

During solidification study, we did observe the presence of local structural order within the supercooled liquid, as detailed in **Supplementary Fig. 20**. Measuring chemical short-range order in a liquid (or amorphous) state is inherently different (less straightforward) than in a crystalline structure. To further clarify this, we adopted the following methodology:

First, we compute the radial distribution function $g(r)$ in the liquid. The liquid regime is divided into 6 slabs according to their distance away from the solidification front (**Supplementary Fig. 21a**). **Supplementary Fig. 21b** shows the corresponding radial pair distribution functions in these six zones. The peaks in these regions show location independence. With the first peak distance, we then compute the number of i-j pairs (i and j type of atom). The corresponding results are shown in **Supplementary Fig. 21c**. The number of chemical pairs in the six regimes remains the same, suggesting there is no strong chemical short-range ordering in the liquid region even close to the solidification front.

Regarding the choice of EAM potential, we conducted additional solidification simulations using a different EAM potential[4]. This was done to validate our conclusion that "prevalent SRO can form during the solidification process, even at high cooling rates." **Supplementary Fig. 22** shows that no significant CSRO forms in the liquid prior to MPEA solidification.

Supplementary Note 4

Our solidification model, using the model CoCrNi EAM potential, showed that Ni-Ni and Co-Cr bonds are favorable. This potential is particularly calibrated to capture the energetics of stacking faults and chemical ordering in the Ni–Co–Cr system. To understand the thermodynamic origin of this ordering, we perform further and detailed analysis.

First, we compute the cohesive energy for all pairs (Ni-Ni, Co-Co, Cr-Cr, Ni-Co, Ni-Cr, Co-Cr), as shown in **Supplementary Table 4**. The corresponding order parameters are also shown in the right column. It can be seen that the most favored pair (Co-Cr) in the ternary alloy does not correspond to the lowest pair energy. We also compute the mixing enthalpy for the binary alloy, Co-Cr, Ni-Co, and Ni-Cr (**Supplementary Table 5**). The Ni-Cr, showing a negative heat mixing, does not result in a favored pairing in the ternary alloy CoCrNi. We note that the classic way of computing mixing enthalpy (or cohesive energy) is using binary alloy, not considering the presence of the third element. Taking CoCrNi as an example, when computing the mixing enthalpy of Ni-Cr, the influence of Co has yet to be considered.

To understand the driving force for Co-Cr and Ni-Ni ordering in ternary CoCrNi, we perform the insertion of Ni atom to Co-Cr alloys and see how the mixing enthalpy varies. **Supplementary Fig. 23** shows the enthalpy changes when increasing the Ni concentration x in the $(CoCr)_{1-x}Ni_x$ alloys. The addition of Ni to CoCr causes an increase in enthalpy, which indicates that CoCr is a favored pair. The ordering of CoCr will leave Ni clustering. **The results suggest that when interpreting the chemical ordering in ternary alloys, the presence of all species for enthalpy calculation may need to be considered**.

Furthermore, we perform simulation and calculation using a different EAM potential[4]. This potential was specifically developed to investigate the phenomena of sluggish diffusion and microtwinning at cryogenic temperatures within the CoCrFeMnNi high-entropy alloy system. The spatial distribution of the order parameter for CoCrNi after annealing at 1,315 K is shown in **Supplementary Fig. 24 a-b**. One can see that this potential gives rise to different results in which Ni-Cr, Co-Co, and Cr-Cr are favorable pairs. The binary mixing enthalpy indicates Ni-Co has the lowest negative value (**Supplementary Table 6**), and again, it could not interpret the ordering in ternary alloy. To understand the influence of the third element, we compute mixing enthalpy as a function of Co concentration x in $Co_x(CrNi)_{1-x}$. As shown in **Supplementary Fig. 24c,** the enthalpy increase with Co implies that Cr-Ni is favored and Co-Cr and Co-Ni are unfavored, which aligns with the measured order parameters in the ternary alloy.

Lastly, we used the 2nd EAM potential[4] and performed solidification modeling. Under the same fast colling rate of 10^{11} K/s, a high degree of chemical short-range order has developed in the solidified region (**Supplementary Fig. 25**). This indicates that the fast atomic diffusion in the supercooled liquid matches or even surpasses the solidification rate and enables chemical ordering at a short timescale. This finding using a different EAM potential agrees with the main conclusion in our manuscript (i.e., “the diffusion of atoms in liquid is fast enough to endow the chemical ordering during rapid solidification”).

The complex interplay between mixing enthalpy and ordering tendency is a fascinating aspect of multicomponent systems that warrants further in-depth investigation.

Supplementary Note 5

Previous work has shown that an increased density of defects, such as stacking fault tetrahedrons and vacancies[5], will develop in the samples with a high cooling rate.

Can the increase in SRO be clearly distinguished from the rise in other defect types and configurations? We would like to address it from two perspectives:

1. Physical process perspective:

It appears that the rise in SRO cannot be entirely isolated from the increase in other defect types and configurations. Recent findings suggest a correlation between SRO and stacking faults. For instance, Naghdi et al. observed a significant augmentation in chemical ordering within stacking fault regions using molecular dynamics simulations[6]. Similarly, experiments by Soel et al. have shown that mechanically induced SRO is directly associated with stacking faults[7]. This evidence supports the notion that the formation of SRO and certain defects may be interrelated phenomena.

2. Characterization perspective:

Experimentally, the precise quantification of SRO remains a formidable challenge. A commentary in *Nature Materials* has indicated that stacking faults can also contribute to diffuse diffraction signals[8]. Moreover, due to the potential correlation between stacking faults and SRO, as noted earlier, a clear separation in characterization seems currently unattainable. Nevertheless, in our experiments, we have endeavored to select regions devoid of stacking faults for SAED analysis to facilitate the differentiation of SRO from defects to some extent. Given that our study's primary objective is to qualitatively assess the presence or absence of SRO in samples with high cooling rates, the precise separation of SRO from defects, while ideal, is not critical for our conclusions.

In simulations, we can precisely characterize SRO after rapid solidification and after annealing, as the atomic position of each element is known. The concordance between experimental results and simulations enhances the credibility of our conclusions.

Supplementary Note 6

Supplementary Fig. 26 shows a comprehensive comparison of the relative SRO intensity in all samples used in this study.

It is well-acknowledged that FIB samples inevitably contain dislocation loops on the surface due to Ga ion irradiation[9,10]. In this work, we employed two methods for TEM sample preparation:

1. **FIB + Flash Polishing:** After sample fabrication by FIB, we used a flash polishing method (detailed in our **Methods** section) to remove the FIB-induced surface damage. The samples in **Figs. 1** and **2** were prepared using this approach.

2. **FIB Only:** This method was utilized for preparing the sample for in-situ TEM mechanical testing shown in **Fig. 5**. Flash polishing was not feasible here as it might compromise the stability of the mechanical testing process by etching the push-to-pull (PTP) structure or altering the crack shape, which is undesirable. The crack was deliberately created to localize the damage zone for a more precise capture of the SRO-deformation relationship.

Consequently, the difference in SRO relative intensity between **Figs. 1** and **5** stems from the distinct TEM sample fabrication methods. The FIB-only sample (**Fig. 5** in the manuscript) exhibits a weaker SRO signal than the flash-polished sample (**Fig. 2** in the manuscript), as the irradiation process can destroy SRO[7]. This finding also highlights that irradiation can be an effective method to tune SRO, which is also mentioned in our discussion in the manuscript.

While the sample used for the in-situ TEM nanomechanical testing contains some level of FIB damage, this does not undermine the reliability and accuracy of our conclusions. FIB damage is known to increase material brittleness[6] or reduce SRO[11]. Nonetheless, our in-situ TEM nanomechanical testing aims to investigate how SRO is affected by mechanical deformation. We compare the SRO in the same sample region before and after loading, thus the initial ductility or SRO level is not critically important.

Supplementary Note 7

The exposure times for the data in **Fig. 1, Fig. 2,** and **Fig. 5** are 1 s, 0.1 s, and 1 s, respectively. Some of the matrix peaks in the data for **Fig. 1** and **Fig. 5** have been over-exposed to enhance the signal-to-noise ratio for the SRO peaks. Note the experiments in **Fig. 1** and **Fig. 5** were performed before the experiments shown in **Fig. 2**.

The use of the relative SRO intensity, which involves matrix peak intensity, may not be necessary for the in-situ TEM experiments. The consistency of the sample area for SAED imaging and the imaging conditions throughout the experiments lends confidence that a straightforward comparison of the absolute SRO intensities is adequate.

We utilized the MRC files from the original data to examine the evolution of the SRO peak intensity. Although some of the matrix peaks are overexposed, all the SRO peak's intensities are properly exposed as their intensities are 20-50 times lower than the matrix. We found that the SRO peak intensity decreases as the loading cycle increases (see **Supplementary Fig. 29**).

Moreover, to substantiate our findings, we computed the ratio of SRO intensity to the overall background—the sum total of pixel intensities in a SAED pattern—and found that the outcomes are consistent with our initial results (see **Supplementary Fig. 29**).

To facilitate the comparison with the data in **Fig. 2** and **Fig. 5**, we have applied a scaling factor α to the SRO intensity, thereby converting the SRO peak intensity to the relative SRO intensity. We first conducted a repeat SAED experiment on the same sample region using a 0.1s exposure time, where the SAED was properly exposed, to obtain the relative SRO intensity, denoted as $I_{\text{0.1 second}}^{\text{1000 cycles}}$. Then, the following formulae are used to calculate the scaling factor α:

$$I_{\text{0.1 second}}^{\text{1000 cycles}} = \frac{SRO_{\text{0.1 second}}^{\text{1000 cycles}}}{Matrix_{\text{0.1 second}}^{\text{1000 cycles}}} \tag{2}$$

$$\alpha = \frac{I_{\text{0.1 second}}^{\text{1000 cycles}}}{SRO_{\text{1 second}}^{\text{1000 cycles}}} \tag{3}$$

where the $Matrix_t^N$ and SRO_t^N are the Matrix and SRO peak intensity obtained from the data taken with an exposure time of *t* for the push-to pull (PTP) sample after *N* deformation cycles, respectively. Then the relative SRO intensity in **Fig. 5** is computed using:

$$I_{\text{1 second}}^{N} = \alpha \times SRO_{\text{1 second}}^{N}, \quad N = 0, 500, 1000. \tag{4}$$

Supplementary Note 8

Anisotropic strain within the samples may contribute to either isotropic or anisotropic broadening of the Bragg peaks in SAED patterns. While such strain could potentially bias the peak intensity measurements, we believe that these factors do not detract from the overarching conclusions of our research for the following two reasons:

1. The experiments were conducted multiple times across different samples, consistently yielding similar results. This suggests that the impact of anisotropic strain is minimal and does not significantly influence the observed trends.

2. The primary goal of this study is to conduct a qualitative assessment of the SRO levels, particularly to identify the presence or absence of SRO in samples formed at high cooling rates. As such, our focus is not on the fully quantitative measure but rather on the qualitative detection of SRO, which remains unaffected by the potential variations due to anisotropic strain. Therefore, the credibility of our characterizations related to the existence of CSRO under the conditions studied is maintained.

To verify if the strain-led peak-broadening effect is ignorable, we performed two further analyses of our data. The schematic (**Supplementary Fig. 30**) shows the definition of variables used in our analyses.

First, we checked the full width at half maximum (FWHM) of the matrix peak for different types of samples (**Supplementary Fig. 31a**). Although it was assumed that the samples with a higher cooling rate, such as the LDED and LPBF samples, may have a higher density of defects, we did not observe a more significant peak-broadening effect in these samples (**Supplementary Fig. 31b**).

Second, we used the integral of the peak to recalculate the relative SRO intensity. This method, using the integral of the peak (as shown in **Supplementary Fig. 30**), calculates the shadow area under the peak curve, which can effectively ensure reliability even with the strain-led peak-broadening effect. The comparison between peak-value-based relative SRO intensity and integral-value-based relative SRO intensity is shown in **Supplementary Fig. 32**. Our new analysis reveals the same conclusion as the one in our previous manuscript, showing that the strain-led peak-broadening has a neglectable effect on our conclusion.

Supplementary Figure

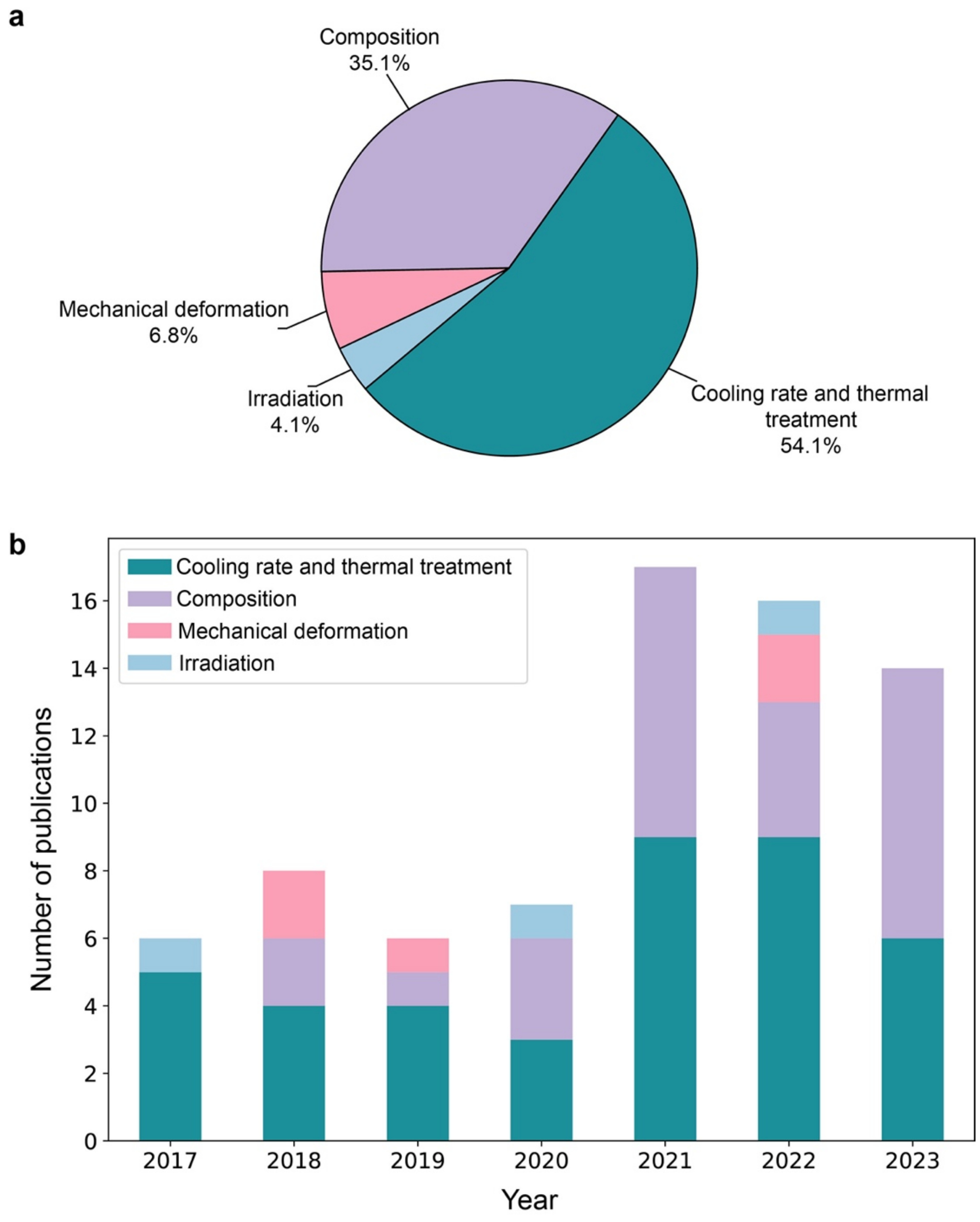

Supplementary Fig. 1 | Comprehensive analysis of literature pertaining to SRO tuning (Jan 2017 - July 2023). a, A pie-chart representation illustrating the proportion of literature related to each SRO tuning method. **b**, A detailed year-by-year breakdown, highlighting the evolution of focus in the field.

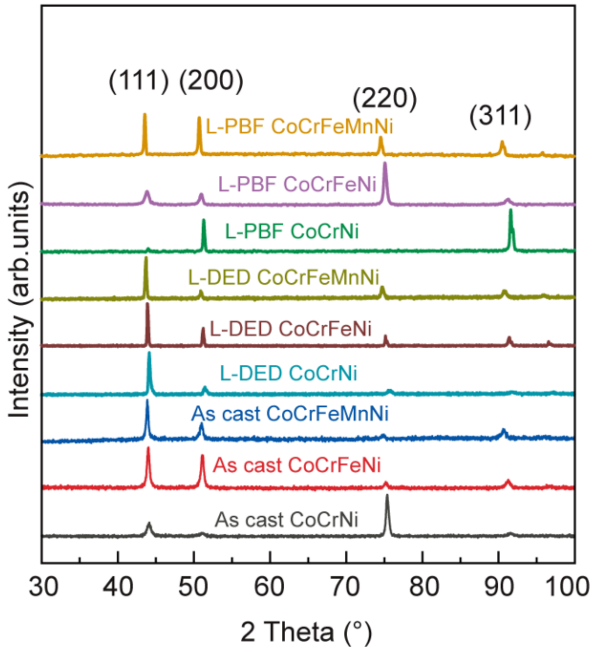

Supplementary Fig. 2 | XRD characterization of the CoCrNi-based MPEAs used in this study.

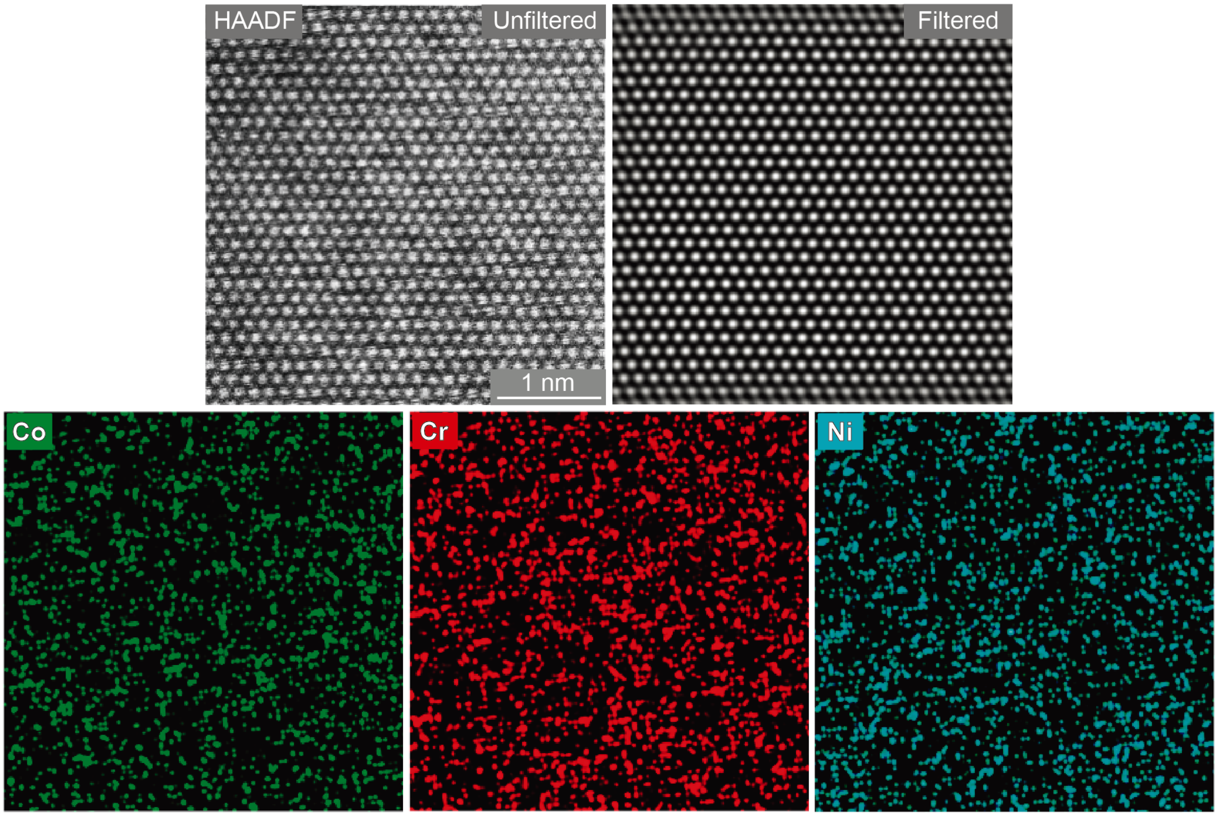

Supplementary Fig. 3 | Atomic resolution STEM-HAADF and EDX mapping for LPBF CoCrNi, captured at [110] zone axis.

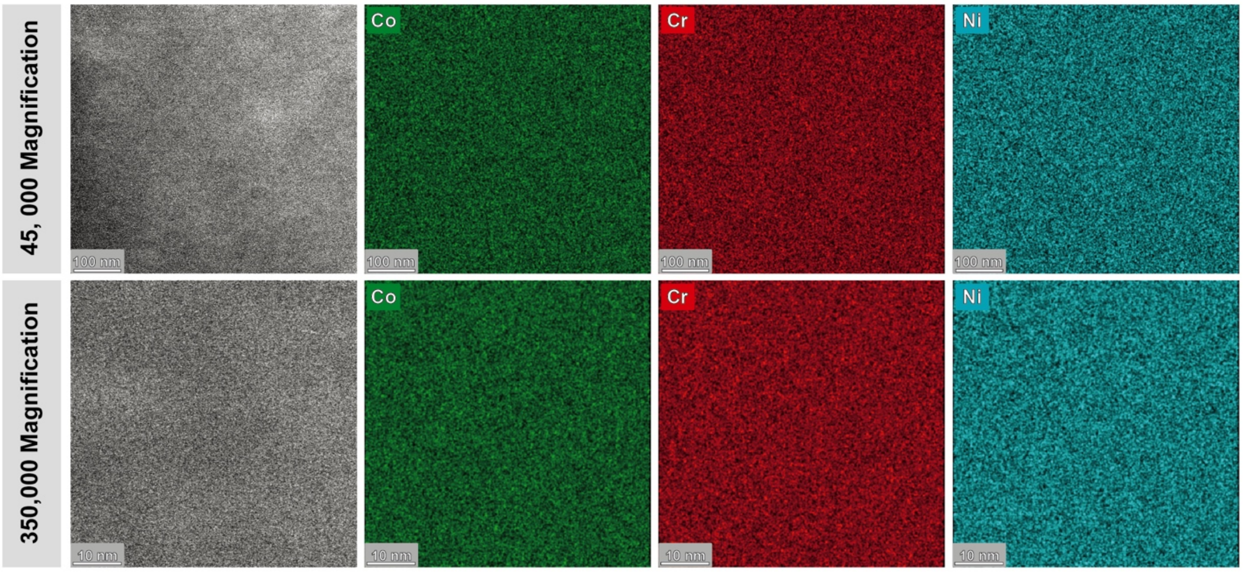

Supplementary Fig. 4 | STEM-EDX mapping for annealed CoCrNi.

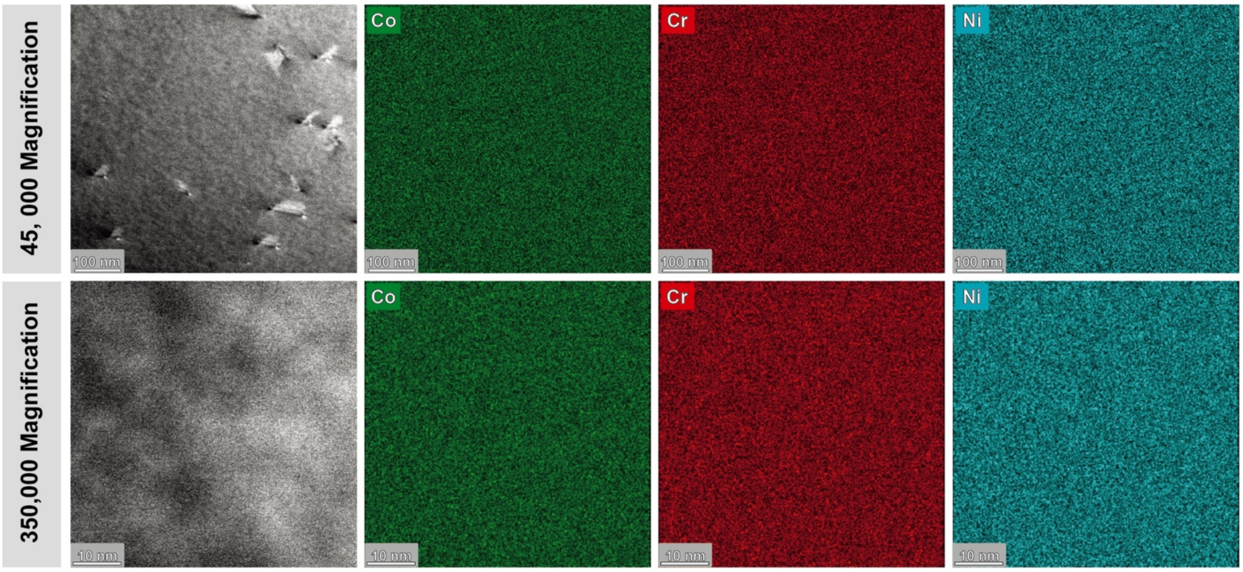

Supplementary Fig. 5 | STEM-EDX mapping for as cast CoCrNi.

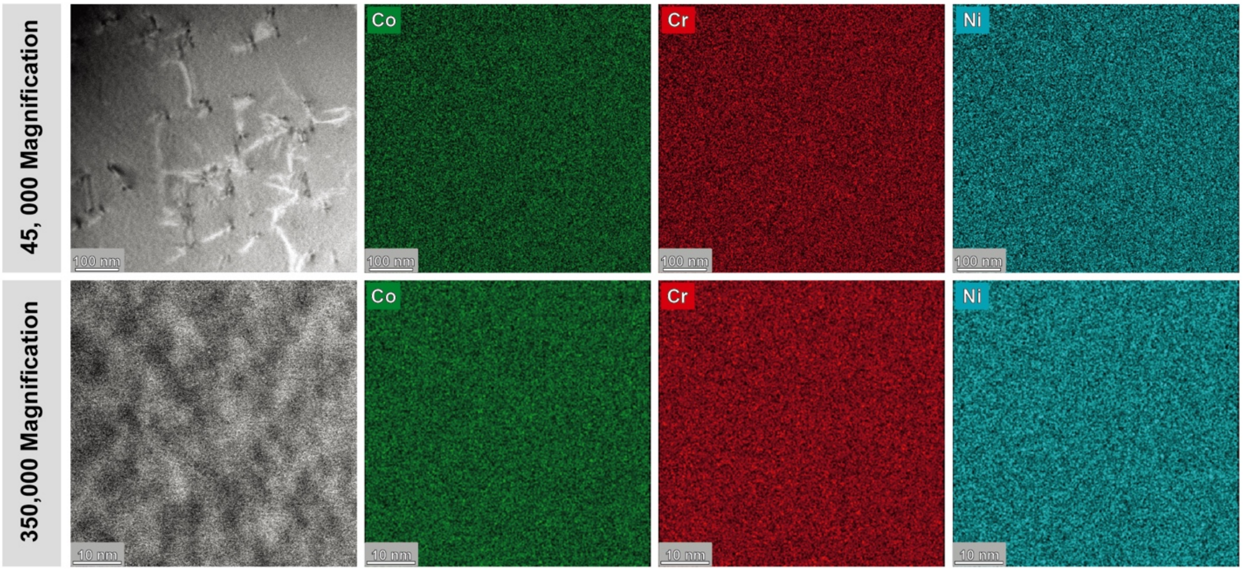

Supplementary Fig. 6 | STEM-EDX mapping for LDED CoCrNi.

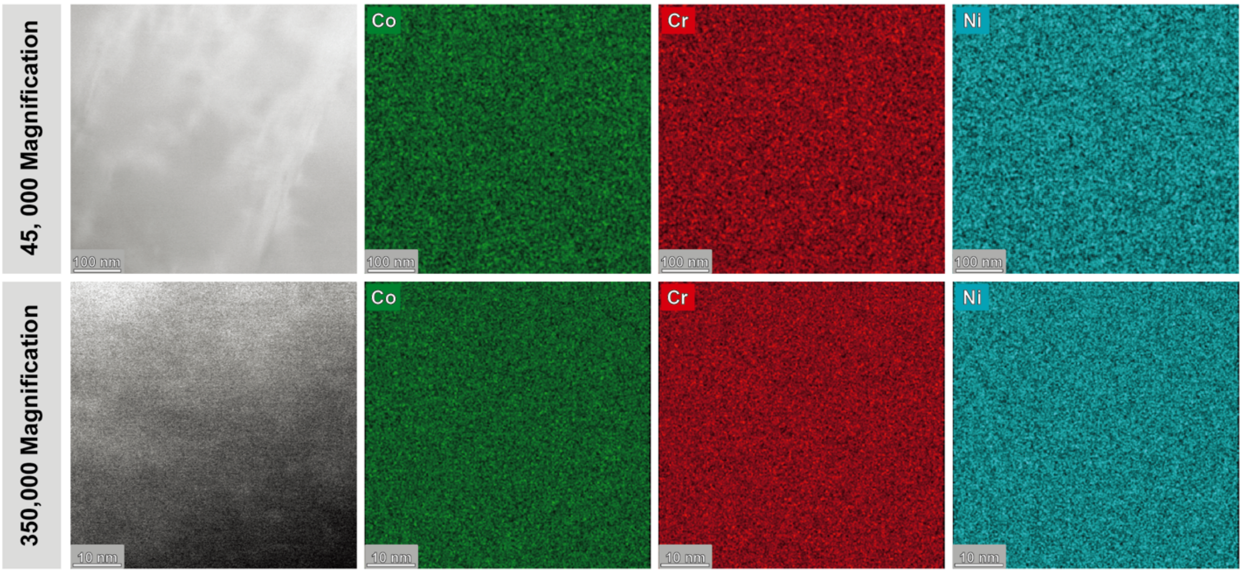

Supplementary Fig. 7 | STEM-EDX mapping for LPBF CoCrNi.

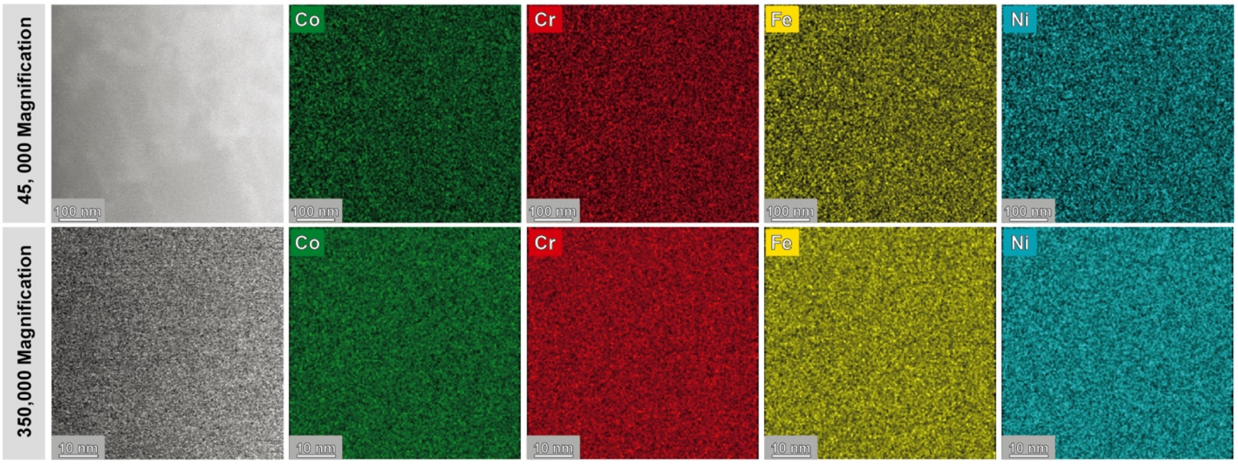

Supplementary Fig. 8 | STEM-EDX mapping for as cast CoCrFeNi.

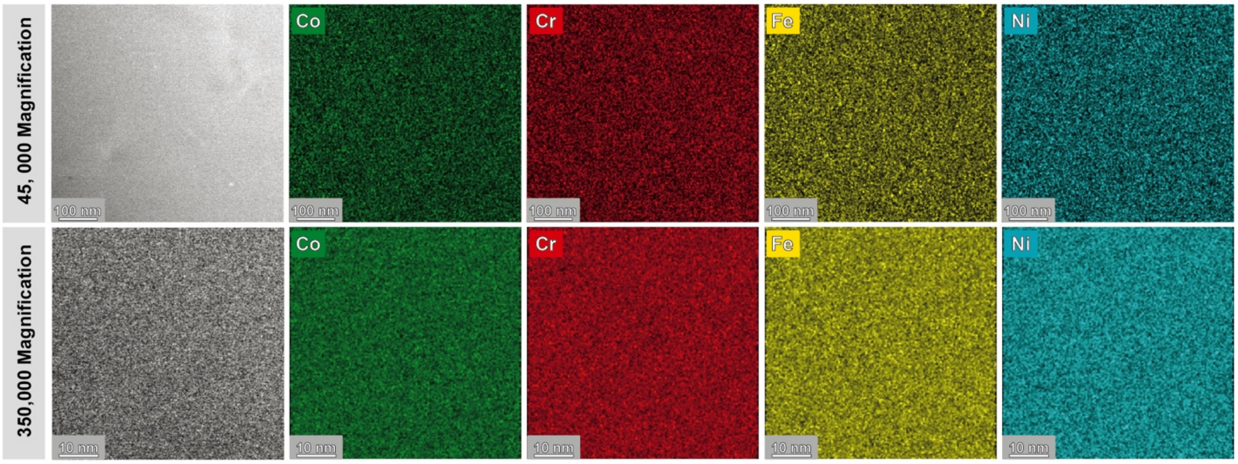

Supplementary Fig. 9 | STEM-EDX mapping for LDED CoCrFeNi.

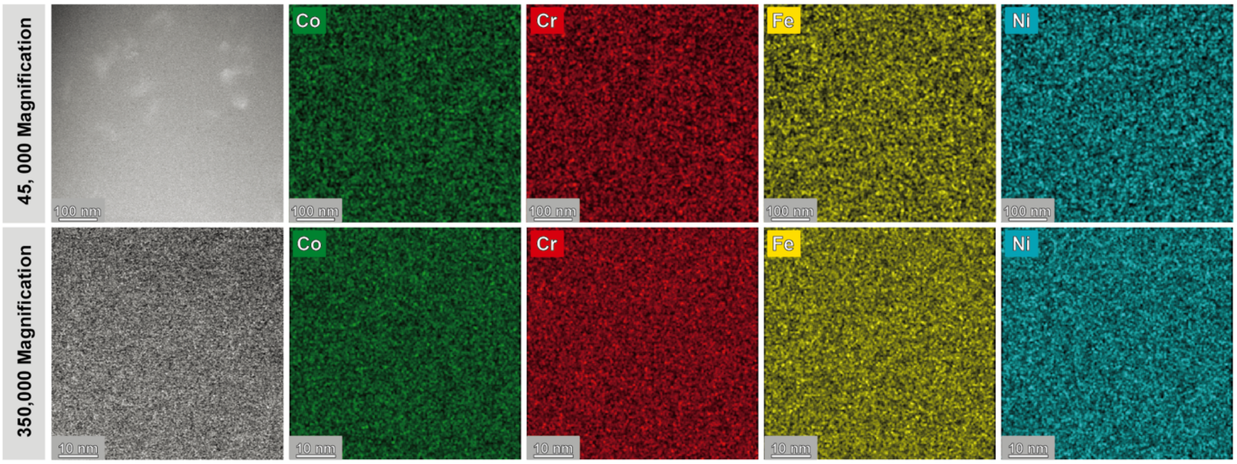

Supplementary Fig. 10 | STEM-EDX mapping for LPBF CoCrFeNi.

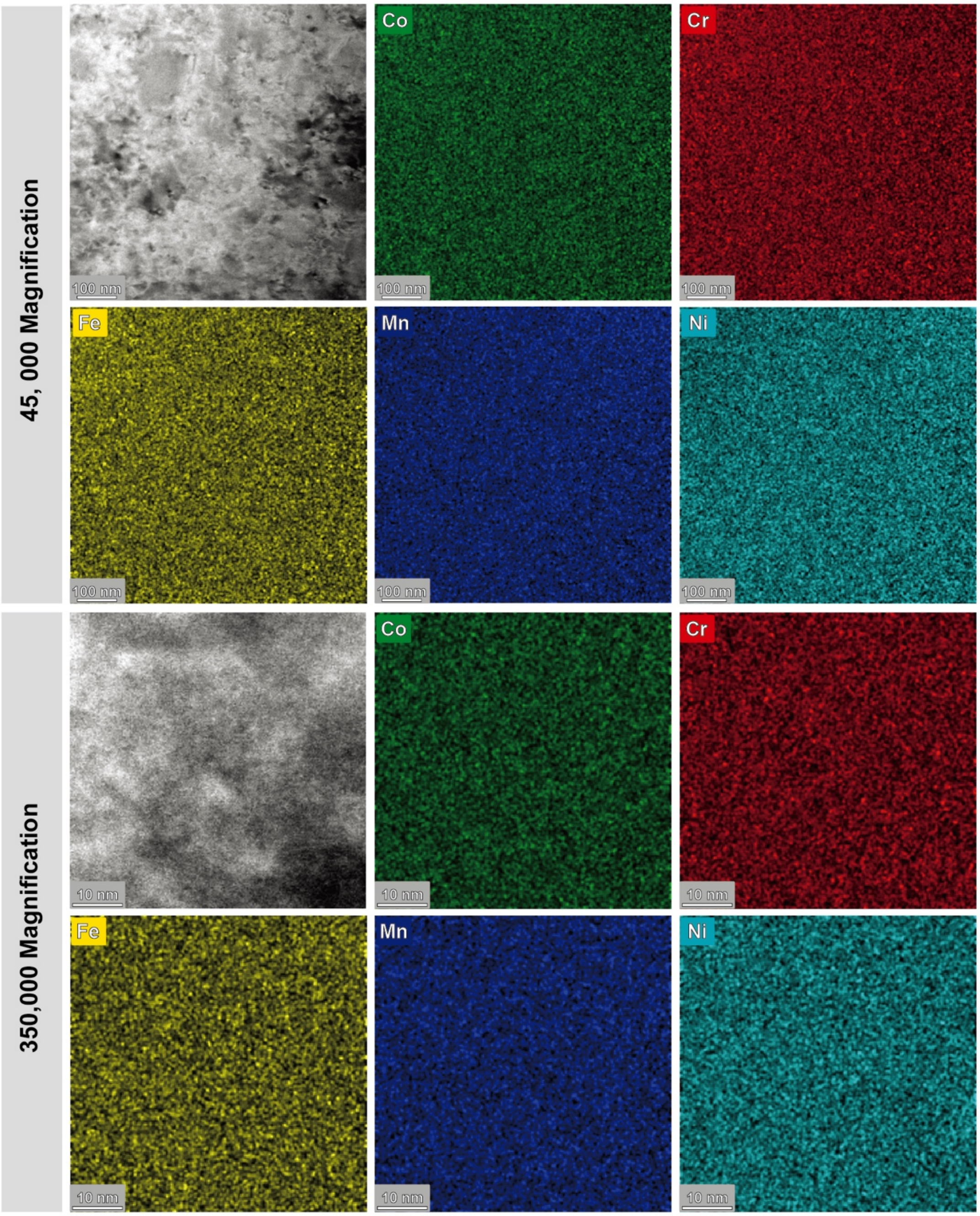

Supplementary Fig. 11 | STEM-EDX mapping for as cast CoCrFeMnNi.

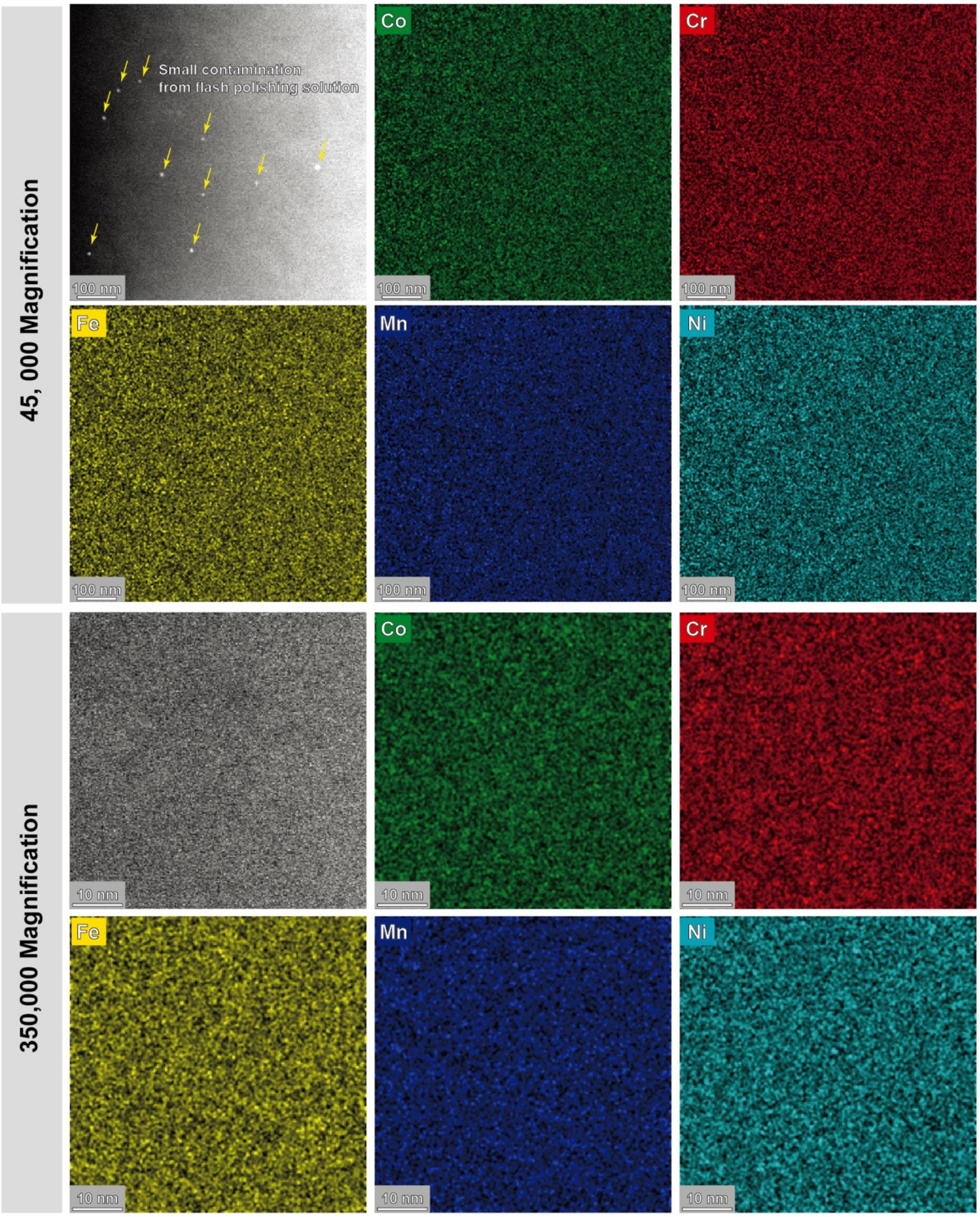

Supplementary Fig. 12 | STEM-EDX mapping for LDED CoCrFeMnNi. These small contaminations are CaO particles.

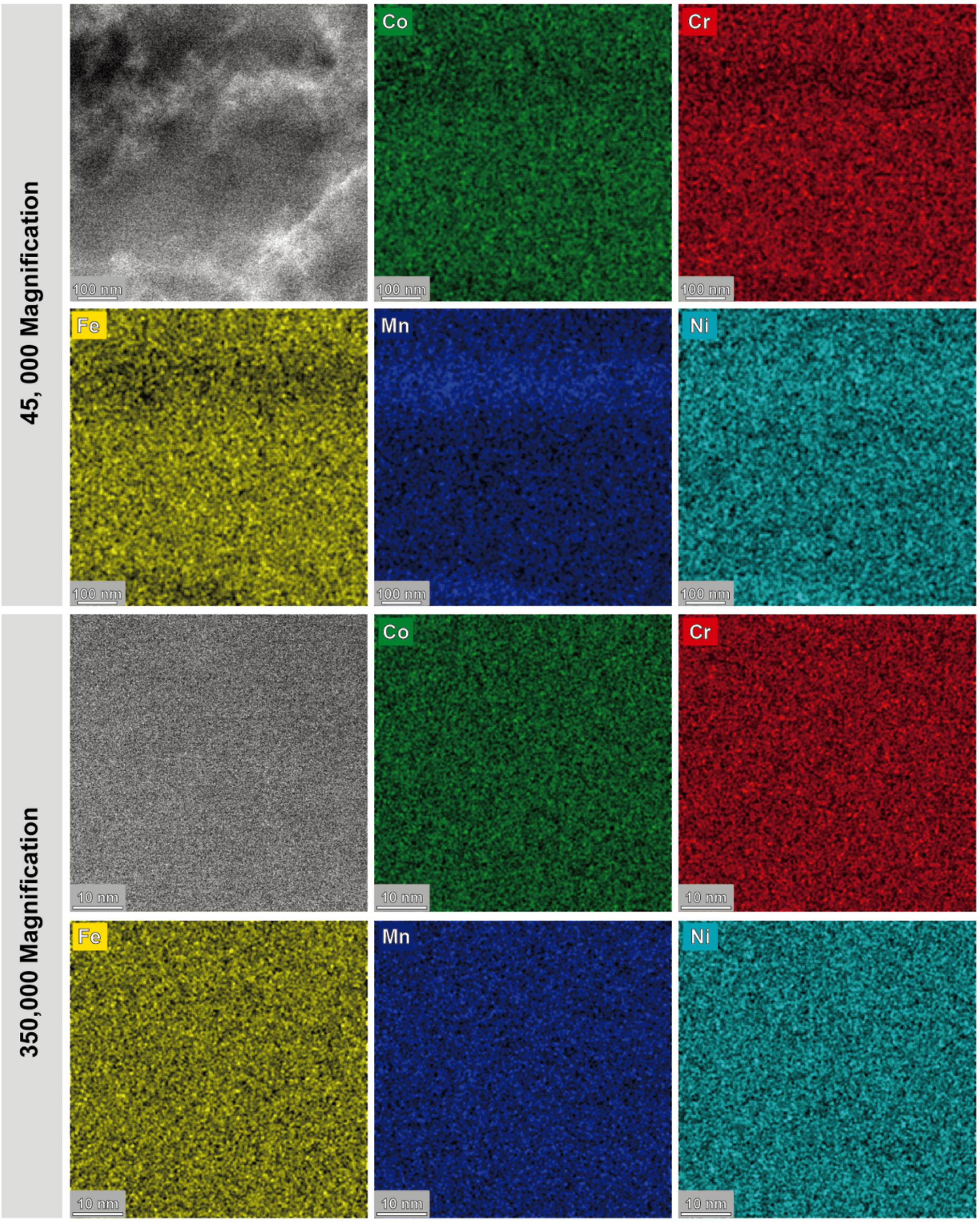

Supplementary Fig. 13 | STEM-EDX mapping for LPBF CoCrFeMnNi.

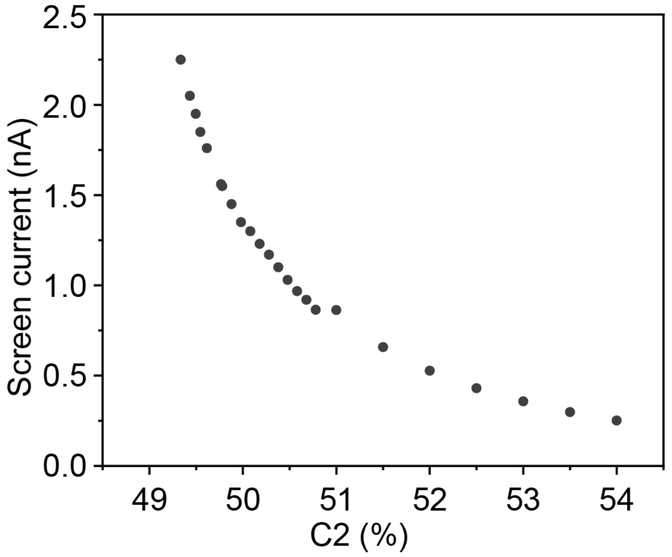

Supplementary Fig. 14 | Measurement of the relationship between C2 and electron beam current. Data was collected at 22,500 × magnification without a select area aperture inserted.

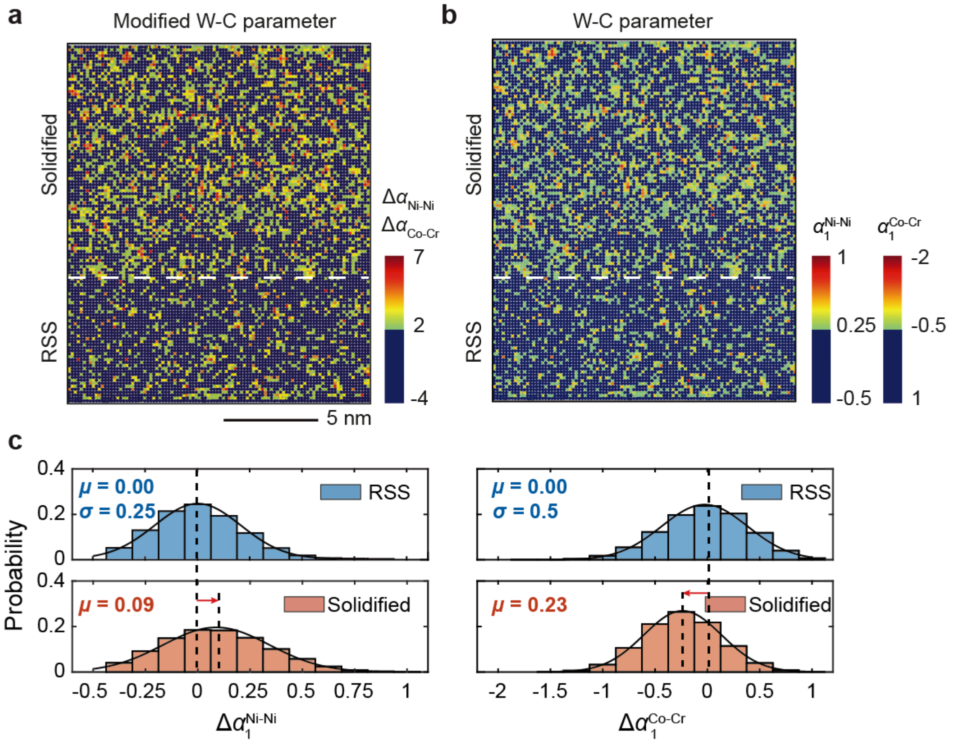

Supplementary Fig. 15 | a, Pairwise order parameter calculated from modified Warren-Cowley parameter, $\Delta\alpha_{i\text{-}j}$. **b,** Pairwise order parameter calculated from Warren-Cowley parameter $\alpha_{m=1}^{ij}$. **c,** Probability distribution of $\alpha_1^{\mathrm{Ni-Ni}}$ and $\alpha_1^{\mathrm{Co-Cr}}$.

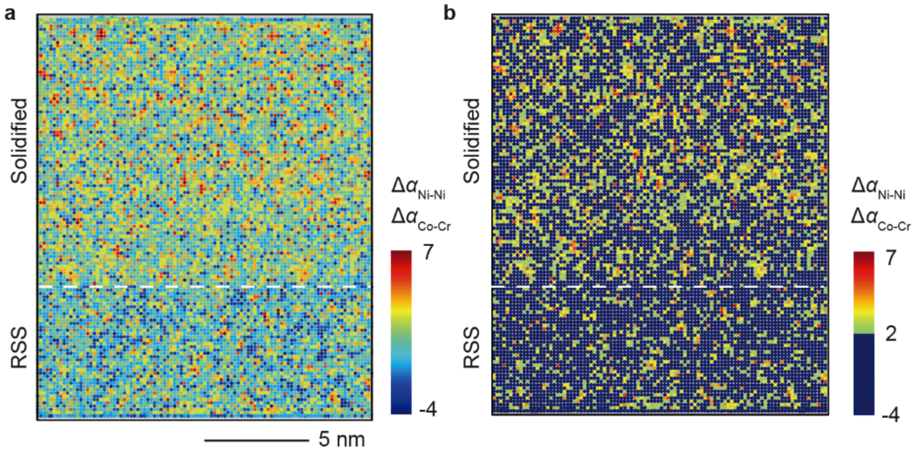

Supplementary Fig. 16 | The spatial distribution of pairwise order parameters $\Delta\alpha_{Ni-Ni}$ and $\Delta\alpha_{Co-Cr}$. a, The original colorbar, which ranges from -4 to 7. **b**, The colorbar with a threshold. Atoms with order parameters smaller than 2 are colored by dark blue.

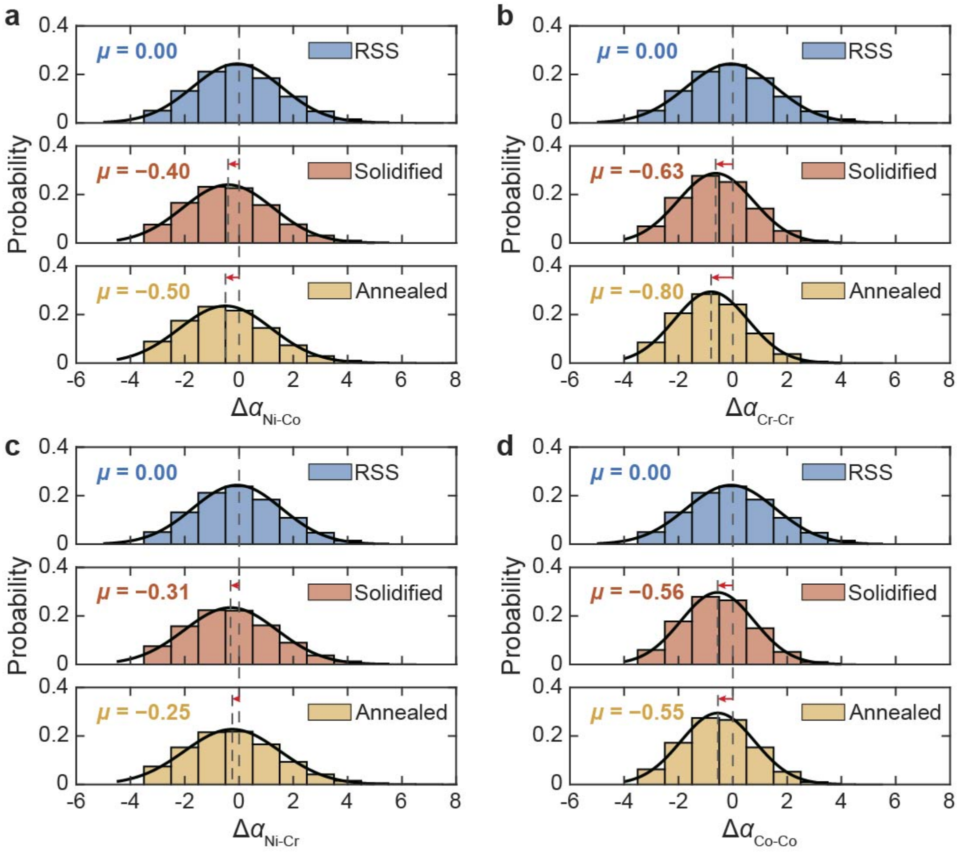

Supplementary Fig. 17 | Modeling the evolution of CSRO during solidification at 1,315 K (continued). a-d, The probability distribution of $\Delta\alpha_{\text{Ni-Co}}$, $\Delta\alpha_{\text{Cr-Cr}}$, $\Delta\alpha_{\text{Ni-Cr}}$ and $\Delta\alpha_{\text{Co-Co}}$.

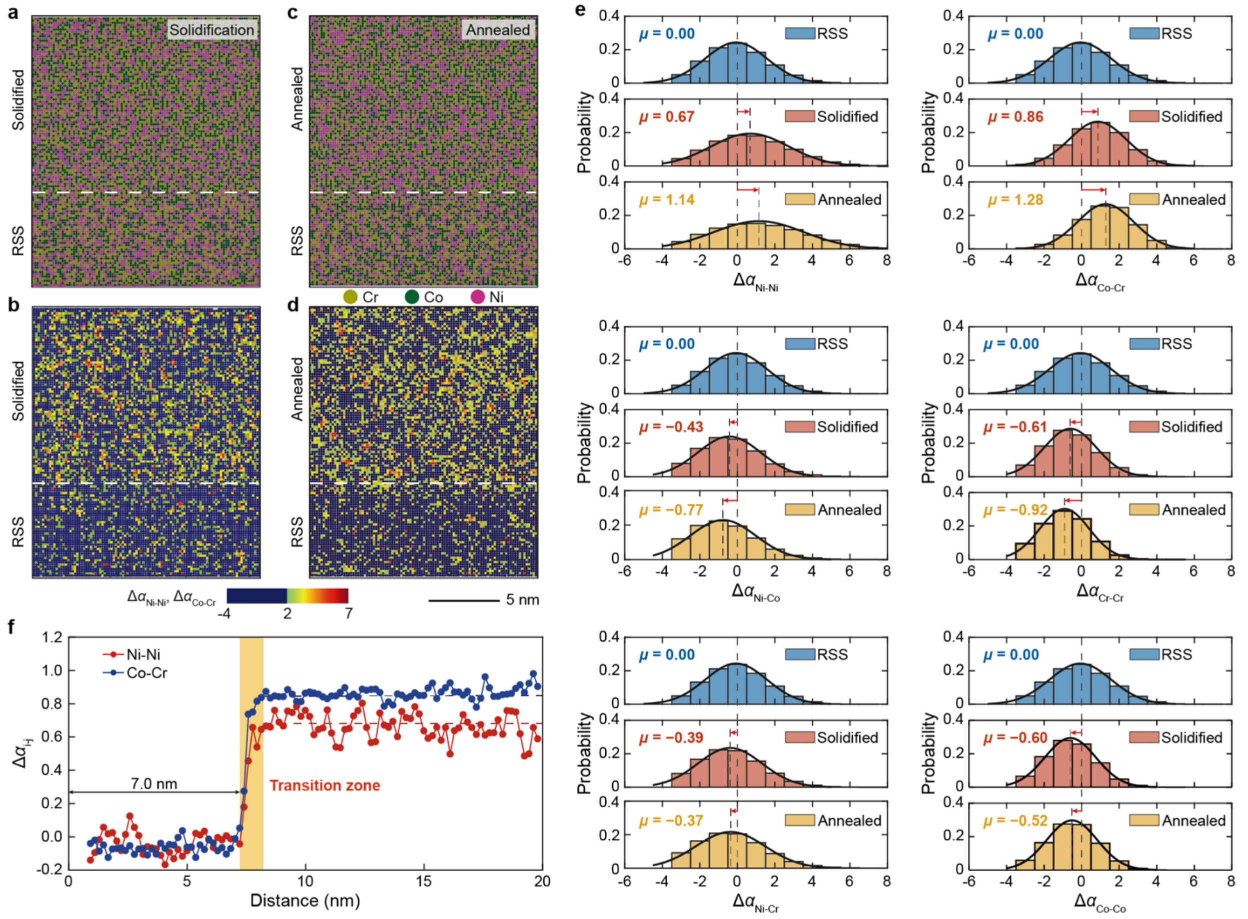

Supplementary Fig. 18 | Modeling the evolution of chemical distribution during solidification and after annealing at 1,015 K. a-d, Chemistry spatial distribution and local pairwise order parameters $\Delta\alpha_{Ni-Ni}$ and $\Delta\alpha_{Co-Cr}$ distribution after solidification (a-b) and annealing (c-d), respectively. **e,** The probability distribution of six pairwise order parameters. **f,** Variation of $\Delta\alpha_{Ni-Ni}$ and $\Delta\alpha_{Co-Cr}$ for each layer as a function of distance along the *z*-axis.

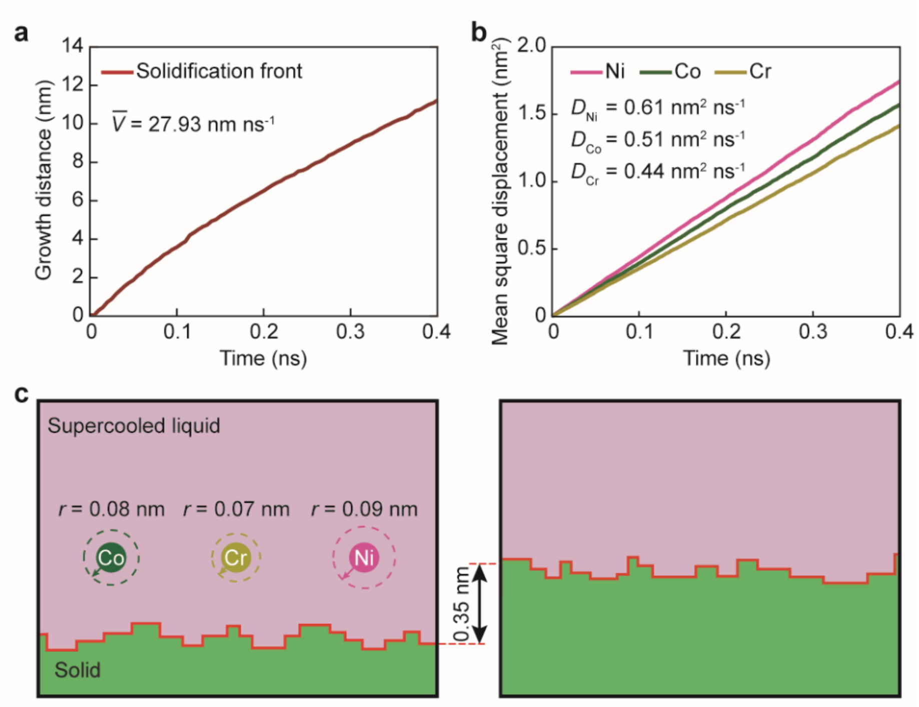

Supplementary Fig. 19 | Comparison of crystal growth distance and diffusivities at 1,015 K. **a**, The growth distance of solidification front as a function of time. The average growth velocity is labeled. **b**, Mean square displacements of elements in the liquid as a function of time. The diffusivities for three elements are labeled. **c-d**, Schematic illustration of growth distance of solidification front and diffusion distance of elements in the liquid at same time.

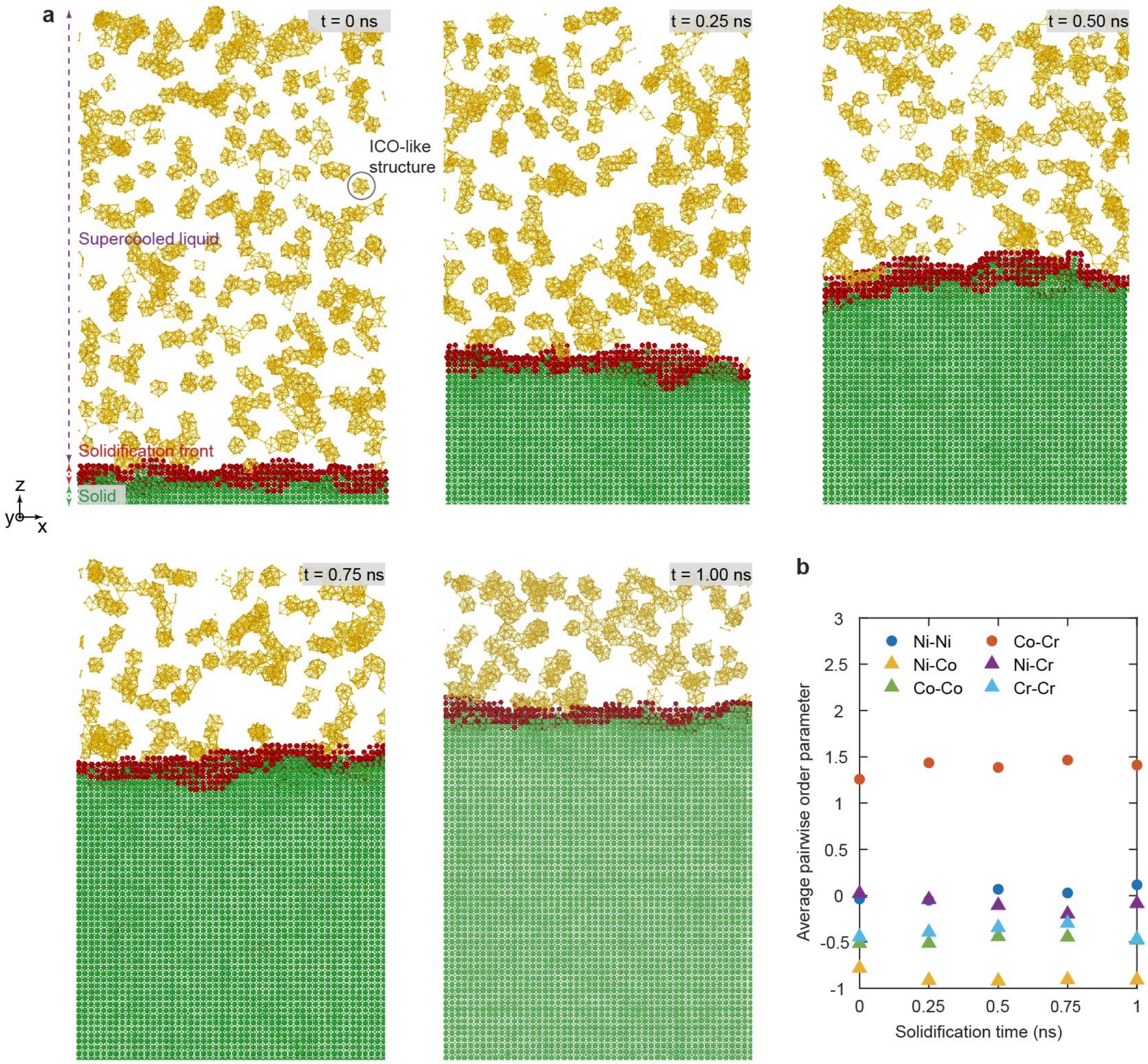

Supplementary Fig. 20 | Analysis of icosahedron structure in the supercooled liquid at 1,315 K during solidification. **a**, Snapshots showing the evolution of the distribution of icosahedral clusters (ICO-like structure) in the supercooled liquid region. **b**, Average pairwise order parameters in ICO-like structure as a function of solidification time.

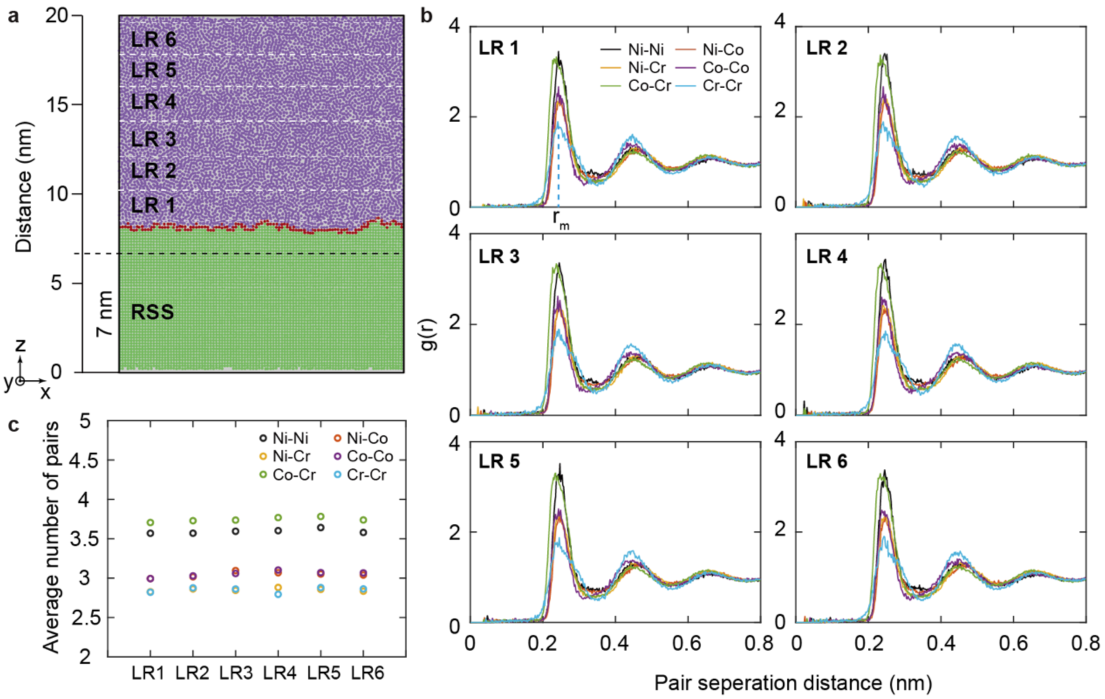

Supplementary Fig. 21 | a, The liquid region (purple colored) in the solidification is divided into six slabs. Their radial pair distribution functions are shown in **b. c,** The number of chemical pairs within the first nearest neighbor as a function of location in the liquid.

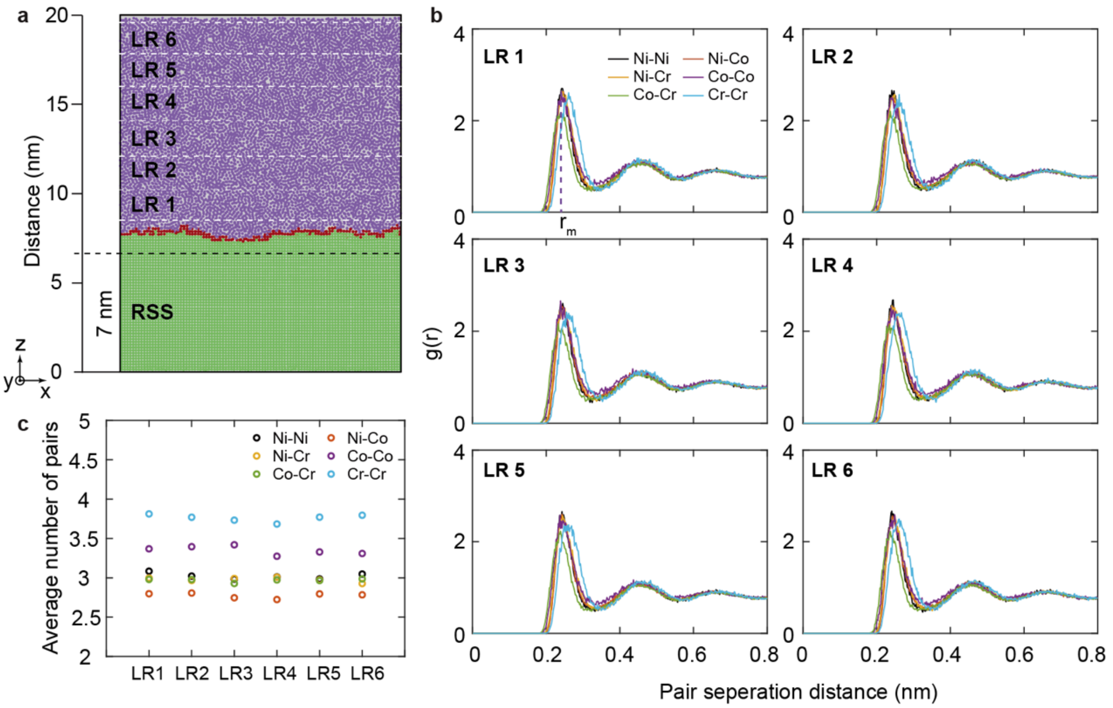

Supplementary Fig. 22 | Similar results using Choi et al. EAM potential. a, The liquid region (purple colored) in the solidification is divided into six slabs. **b,** Radial pair distribution functions of six slabs. **c,** The number of chemical pairs within nearest neighbor as a function of location in the liquid.

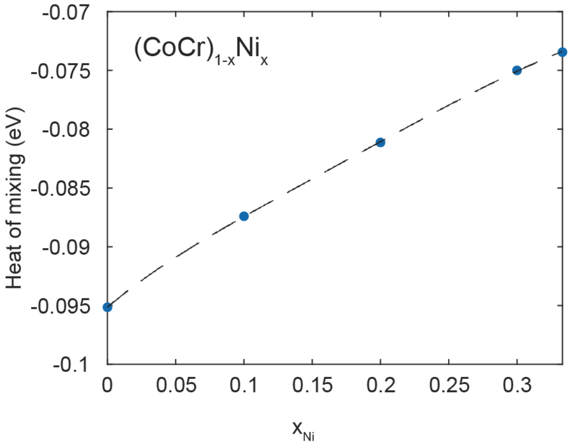

Supplementary Fig. 23 | The effect of adding Ni to the CoCr on mixing enthalpy. The increase of Ni concentration x increases the enthalpy of $(CoCr)_{1-x}Ni_x$ system.

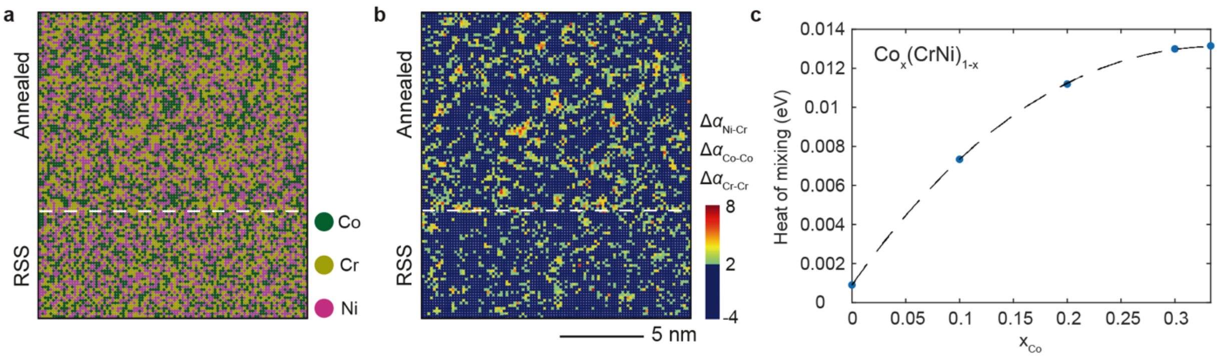

Supplementary Fig. 24 | a, b. Atomic and pairwise order parameters in the annealed CoCrNi alloy using Choi et al. EAM potential. It is noted that Ni-Cr, Co-Co, and Cr-Cr are preferred pairings. **c,** Adding Co increases the enthalpy of $Co_x(CrNi)_{1-x}$ system.

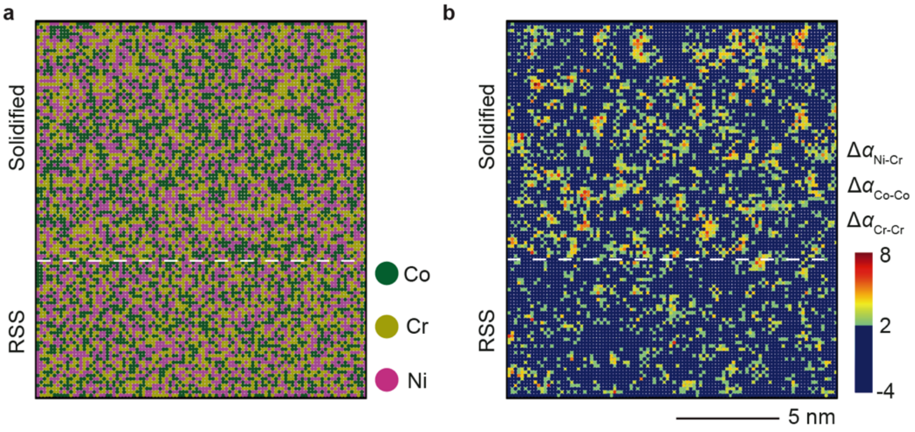

Supplementary Fig. 25 | a, b. Atomic slices of the structure showing the distribution of atoms and pairwise order parameters after solidification, respectively. The results used an EAM potential different from that used for the main figures in this paper.

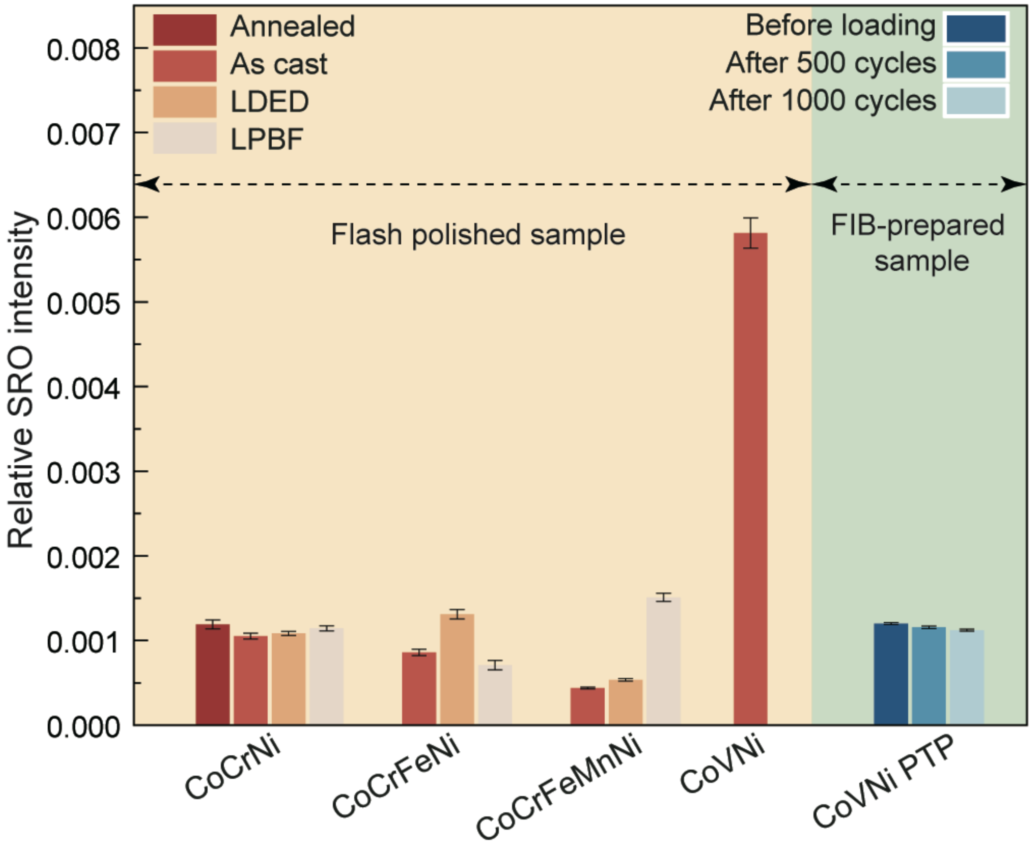

Supplementary Fig. 26 | A comparison of all the samples used in our manuscript. The error bar represents the standard deviation.

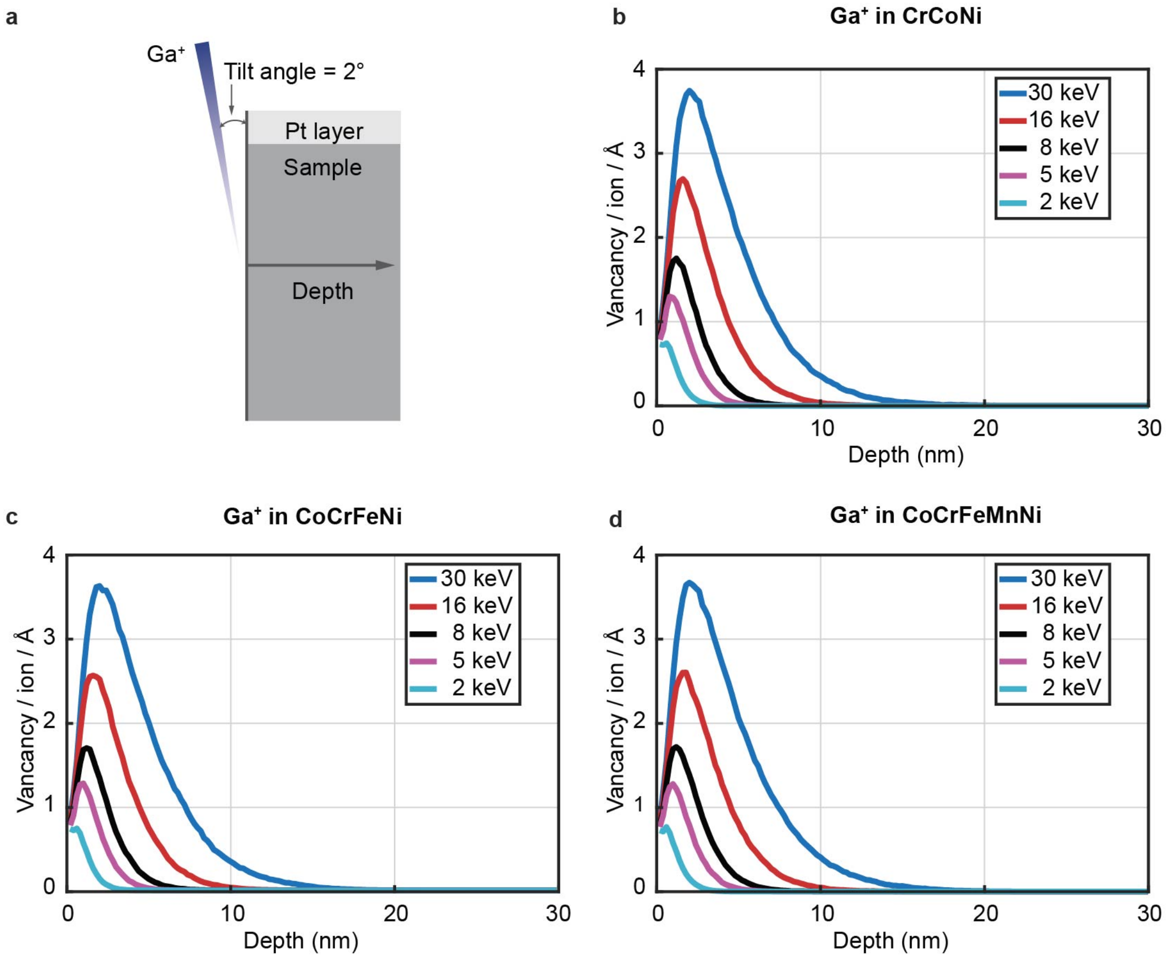

Supplementary Fig. 27 | IM3D simulations to predict the thickness of the damaged layer caused by FIB thinning. a, Schematic drawing of the simulation setup. **b-d**, Vacancy depth-distributions in bulk CoCrNi, CoCrFeNi and CoCrFeMnNi under the irradiation of Ga^{+} at different energies.

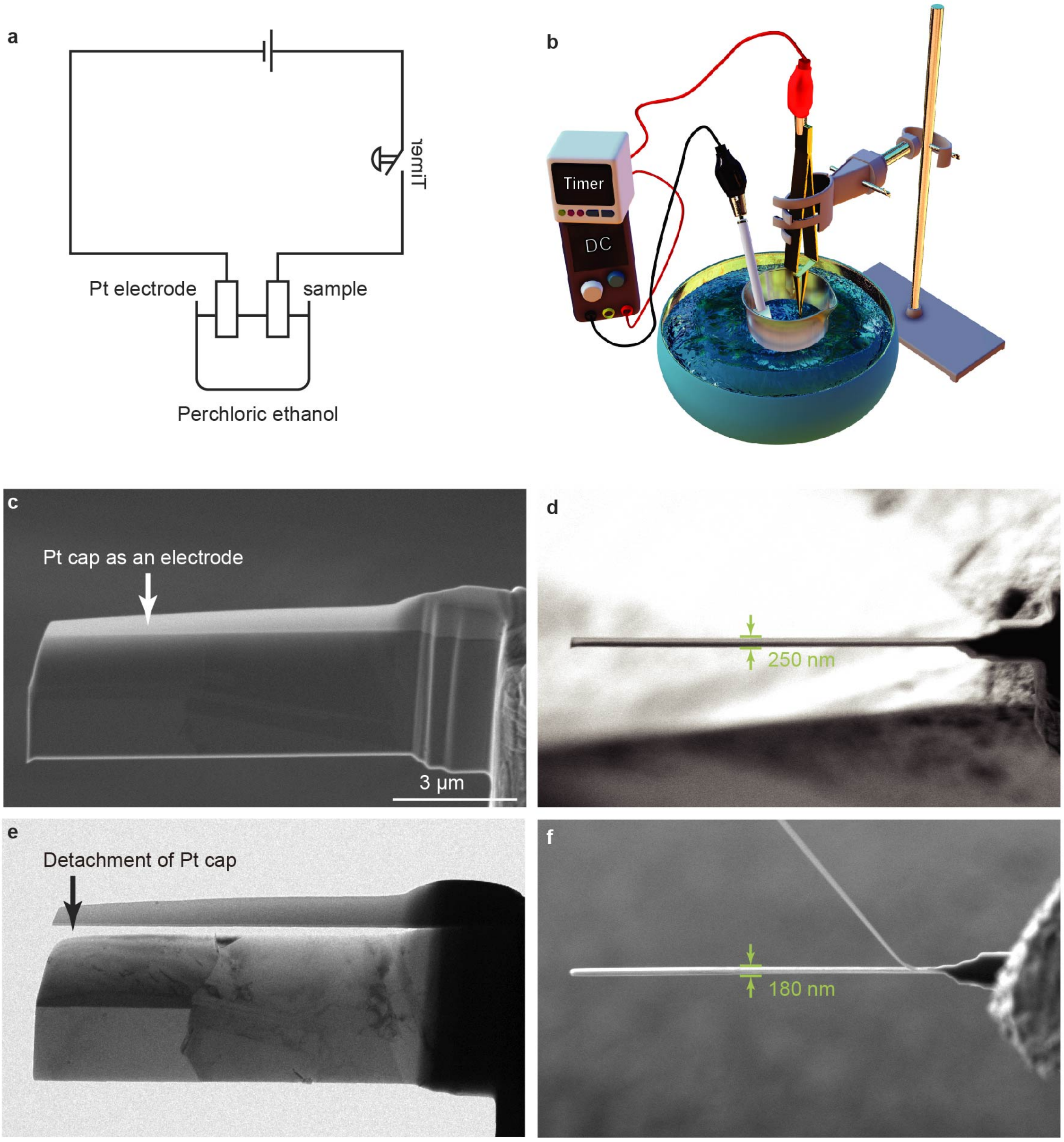

Supplementary Fig. 28 | **a**, Circuit diagram of electrochemical flash polishing. **b**, Schematic of electrochemical flash polishing. **c**,**d**, SEM images before flash polishing. **e**,**f**, TEM and SEM images after flash polishing. Scale bars, 3 µm.

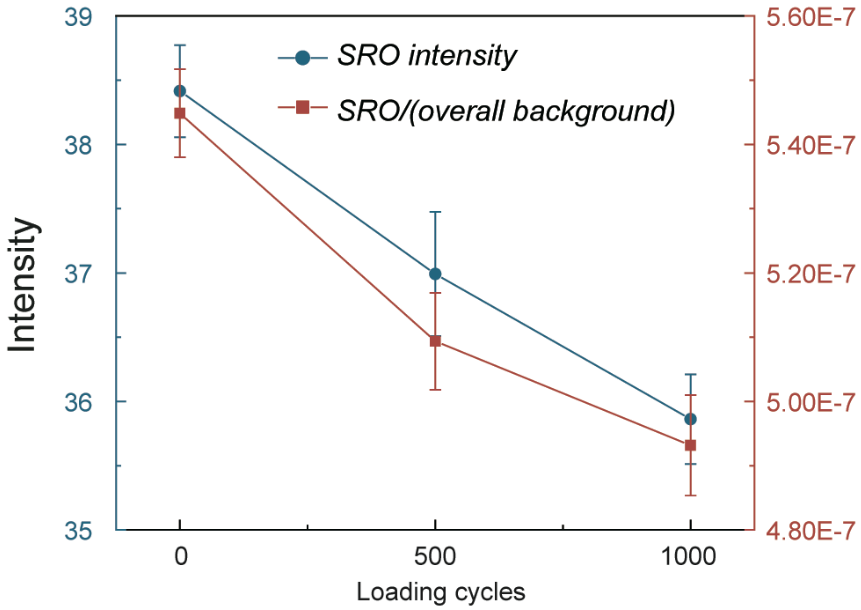

Supplementary Fig. 29 | The SRO intensity and SRO/overall background ratio of PTP sample in Fig. 5. The error bars represent the standard deviation.

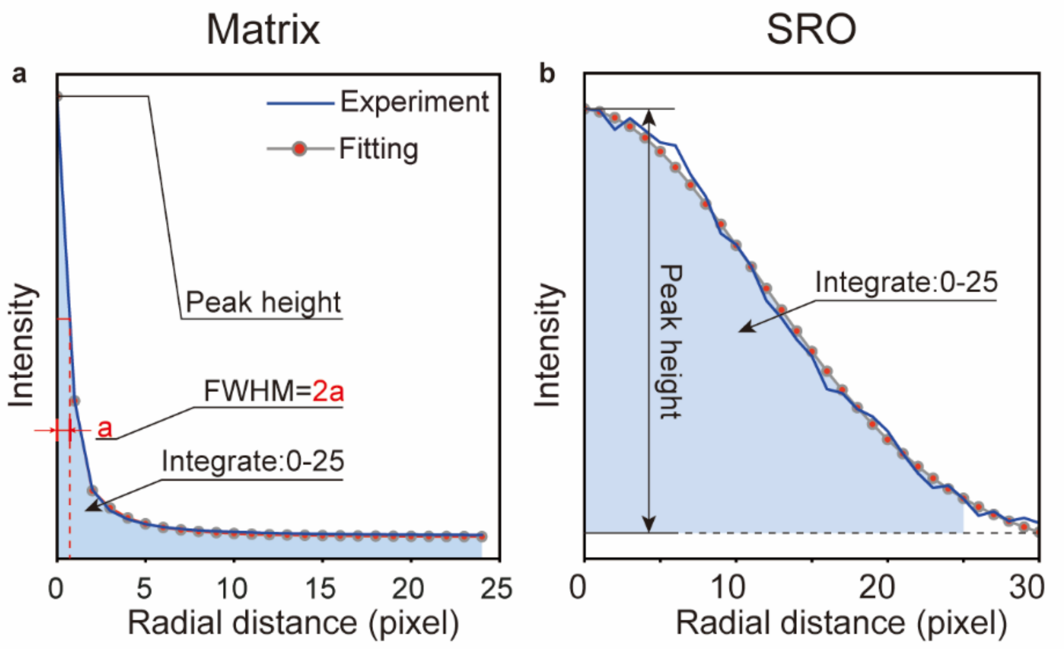

Supplementary Fig. 30 | Schematic showing the definition of variables used in our analyses.

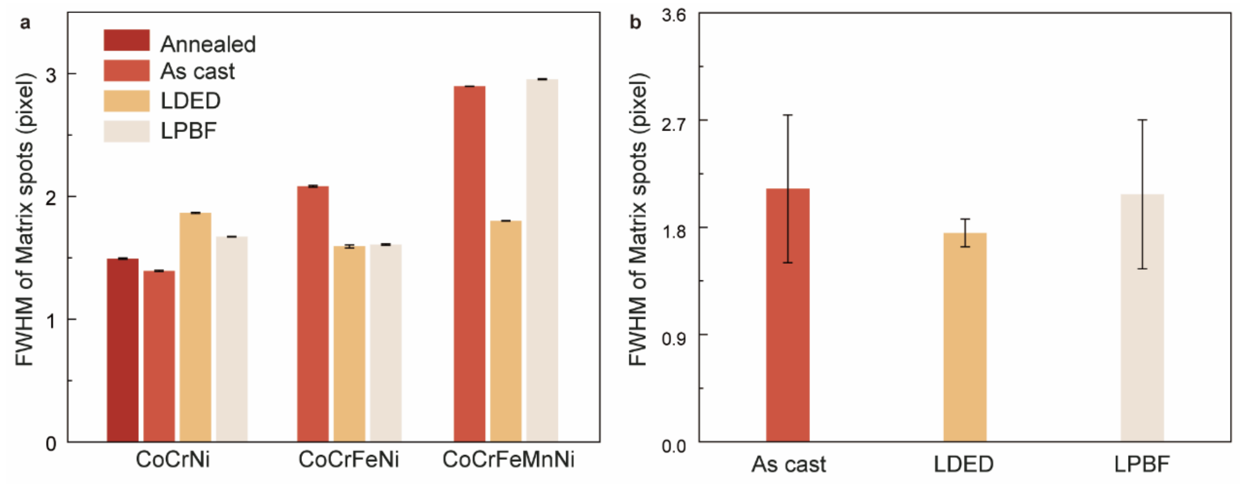

Supplementary Fig. 31 | Full width at half maximum (FWHM) analysis for matrix peaks. a. Averaged FWHM of the matrix Bragg peak in different samples. **b.** Average of matrix FWHM for samples with different cooling rate. The error bars represent the standard deviation.

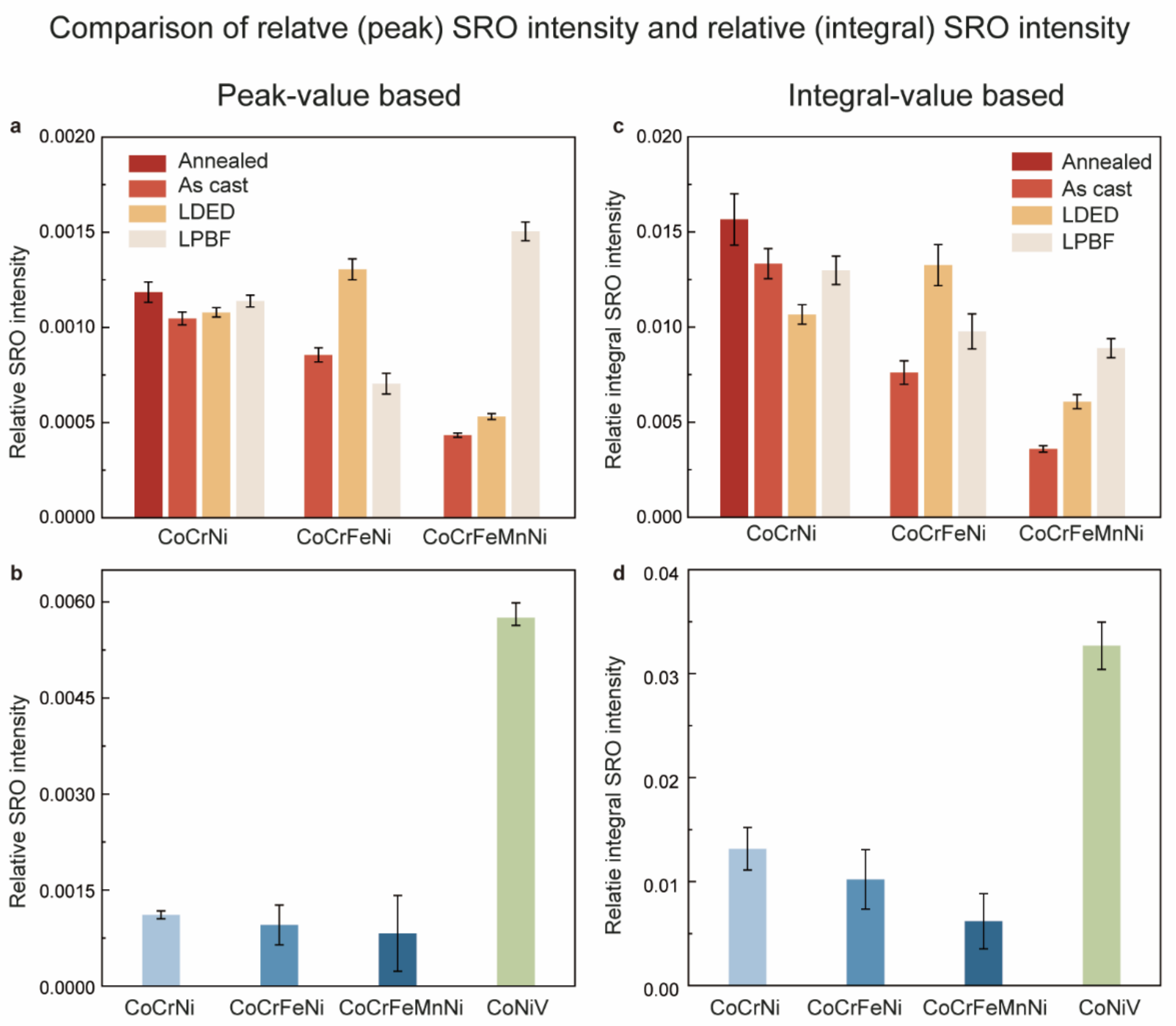

Supplementary Fig. 32 | The comparison between peak-value-based relative SRO intensity and integral-value-based relative SRO intensity. a-b. Results of peak-value-based relative SRO intensity, i.e., the results shown in Fig. 2 in our paper. **c-d.** Integral-value-based relative SRO intensity. The error bars represent the standard deviation.

Supplementary References

1. Li, Q. J., Sheng, H. & Ma, E. Strengthening in multi-principal element alloys with local-chemical-order roughened dislocation pathways. *Nat. Commun.* **10**, 3563 (2019).
2. De Fontaine, D. The number of independent pair-correlation functions in multicomponent systems. *J. Appl. Crystallogr.* **4**, 15-19 (1971).
3. Owen, L. R., Playford, H. Y., Stone, H. J. & Tucker, M. G. A new approach to the analysis of short-range order in alloys using total scattering. *Acta Mater.* **115**, 155-166 (2016).
4. Choi, W. M., Jo, Y. H., Sohn, S. S., Lee, S. & Lee, B. J. Understanding the physical metallurgy of the CoCrFeMnNi high-entropy alloy: An atomistic simulation study. *npj Comput. Mater.* **4**, 1 (2018).
5. Takamura, J. Quenched-in vacancies and quenching strains in gold. *Acta Metall.* **9**, 547-557 (1961).
6. Naghdi, A. H., Karimi, K., Poisvert, A. E., Esfandiarpour, A., Alvarez, R., Sobkowicz, P., Alava, M. & Papanikolaou, S. Dislocation plasticity in equiatomic NiCoCr alloys: Effect of short-range order. *Phys. Rev. B* **107**, 094109 (2023).
7. Seol, J. B., Ko, W.-S., Sohn, S. S., Na, M. Y., Chang, H. J., Heo, Y.-U., Kim, J. G., Sung, H., Li, Z., Pereloma, E. & Kim, H. S. Mechanically derived short-range order and its impact on the multi-principal-element alloys. *Nat. Commun.* **13**, 6766 (2022).
8. Walsh, F., Zhang, M., Ritchie, R. O., Minor, A. M. & Asta, M. Extra electron reflections in concentrated alloys do not necessitate short-range order. *Nat. Mater.* **22**, 926-929 (2023).
9. Kiener, D., Motz, C., Rester, M., Jenko, M. & Dehm, G. FIB damage of Cu and possible consequences for miniaturized mechanical tests. *Mater. Sci. Eng. A* **459**, 262-272 (2007).
10. Liu, J., Niu, R., Gu, J., Cabral, M., Song, M. & Liao, X. Effect of ion irradiation introduced by focused ion-beam milling on the mechanical behaviour of sub-micron-sized samples. *Sci. Rep.* **10**, 10324 (2020).
11. Arkoub, H. & Jin, M. Impact of chemical short-range order on radiation damage in Fe-Ni-Cr alloys. *Scr. Mater.* **229**, 115373 (2023).
